# Mass spectrometry imaging of biomarker lipids for phagocytosis and signalling during focal cerebral ischaemia

**DOI:** 10.1038/srep39571

**Published:** 2016-12-22

**Authors:** Mette M. B. Nielsen, Kate L. Lambertsen, Bettina H. Clausen, Morten Meyer, Dhaka R. Bhandari, Søren T. Larsen, Steen S. Poulsen, Bernhard Spengler, Christian Janfelt, Harald S. Hansen

**Affiliations:** 1 Department of Drug Design and Pharmacology, University of Copenhagen, Universitetsparken 2, DK-2100 Copenhagen, Denmark; 2 Department of Neurobiology Research, University of Southern Denmark, J. B. Winsløws Vej 21, DK-5000, Odense, Denmark; 3 Institute of Inorganic and Analytical Chemistry, Justus Liebig University, Heinrich-Buff-Ring 17, D-35392, Giessen, Germany; 4 National Research Centre for the Working Environment, Lersø Parkallé 105, DK-2100, Copenhagen, Denmark; 5 Department of Biomedical Sciences, University of Copenhagen, Blegdamsvej 3, DK-2200, Copenhagen, Denmark; 6 Department of Pharmacy, University of Copenhagen, Universitetsparken 2, DK-2100, Copenhagen, Denmark

## Abstract

Focal cerebral ischaemia has an initial phase of inflammation and tissue injury followed by a later phase of resolution and repair. Mass spectrometry imaging (desorption electrospray ionization and matrix assisted laser desorption ionization) was applied on brain sections from mice 2 h, 24 h, 5d, 7d, and 20d after permanent focal cerebral ischaemia. Within 24 h, *N*-acyl-phosphatidylethanolamines, lysophosphatidylcholine, and ceramide accumulated, while sphingomyelin disappeared. At the later resolution stages, bis(monoacylglycero)phosphate (BMP(22:6/22:6)), 2-arachidonoyl-glycerol, ceramide-phosphate, sphingosine-1-phosphate, lysophosphatidylserine, and cholesteryl ester appeared. At day 5 to 7, dihydroxy derivates of docosahexaenoic and docosapentaenoic acid, some of which may be pro-resolving mediators, e.g. resolvins, were found in the injured area, and BMP(22:6/22:6) co-localized with the macrophage biomarker CD11b, and probably with cholesteryl ester. Mass spectrometry imaging can visualize spatiotemporal changes in the lipidome during the progression and resolution of focal cerebral inflammation and suggests that BMP(22:6/22:6) and *N*-acyl-phosphatidylethanolamines can be used as biomarkers for phagocytizing macrophages/microglia cells and dead neurones, respectively.

Mass spectrometry imaging (MSI) has increasingly been used to visualize abundance of various molecular species of phospholipids in the tissues^1^,^2^, including neuronal tissues^3^,^4^. This technique holds a great potential to visualize and identify complicated biological processes and cell movements in time and space during localized changes in a tissue, e.g. during inflammation initiation and resolution following focal cerebral ischaemia. Especially, we have hypothesized that the increased presence of the uncommon phospholipids, bis(monoacylglycero)phosphate (BMP)^5^ and *N*-acyl-phosphatidylethanolamine (NAPE)^6^, in brain tissue could be used as biomarkers of phagocytizing macrophages/microglia cells and dead/dying neurones, respectively. Furthermore, it should be possible to visualize the time course of generation of a number of lipid mediators promoting and resolving inflammation in mouse brains exposed to permanent middle cerebral artery occlusion (pMCAO). Focal cerebral ischaemia is characterized with an early ischaemic core surrounded by a penumbra through which the infarct is growing within the first couple of hours after the ischaemic insult^7^,^8^. The first 24 h or more includes an early inflammatory phase involving neutrophil infiltration and release of cytokines and eicosanoids, but after several days it is followed by resolution involving phagocytosis of apoptotic cells and cell debris by macrophages/microglia cells and regeneration^9^,^10^. However, MSI of the progression of these processes with focus on lipid biomarkers and abundance of signalling lipid mediators have not been investigated before.

MSI has already illustrated that during the initial ischaemic phase lysophosphatidylcholine (LysoPC) will accumulate in the ischaemic area^11^,^12^,^13^,^14^, as well as a cessation of Na^+^/K^+^-ATPase activity during cell death can be visualized as a decrease in the abundance of the potassium adduct of intracellular phosphatidylcholine (e.g. PC(34:1)) and an increase in the abundance of the sodium adduct of the same PC species^13^,^14^. Furthermore, ceramides (Cer), *N*-acyl-ethanolamines (NAE), free fatty acids, prostaglandins, and 2-arachidonoylglycerol have been seen by MSI to accumulate in the ischaemic area during the early phase of ischaemia. At the same time sphingomyelin (SM) decreases in abundance^12^,^13^,^14^,^15^,^16^. We have now searched for the spatiotemporal abundance of other PC species, especially docosahexaenoic-containing PC (e.g. PC(18:0/22:6)) and arachidonic-containing PC (e.g. PC(18:0/20:4)), since they have been suggested to be biomarkers for neurones and invasive immune cells, respectively^12^. Since we observed a change in the Na^+^/K^+^-adduct abundance of several PC species, we also visualized the abundance of the Na^+^/K^+^-adducts of sphingomyelin (SM(d18:1/18:0)), because it is generally considered to be localized mainly on the outer leaflet of cells, thereby facing a high sodium concentration^17^. We have previously reported that NAPE accumulates during brain ischaemia in the injured area^13^,^18^,^19^,^20^, and studies of primary cerebral cell cultures suggest that NAPE particularly is generated in dying/dead neurones and not in astrocytes^21^,^22^. Thus, we have used NAPE as a spatiotemporal biomarker for the abundance of dead neurones in the ischaemic brain. BMP is a low abundant phospholipid in all cells being confined to the endosomal/lysosomal vesicles^5^,^23^, but it is especially abundant in macrophages/microglia cells^24^, where it is primarily localized to phagosomes^25^. We have investigated whether BMP can be used as a biomarker phospholipid for macrophages/microglia cells performing phagocytosis during the repair phase of focal cerebral ischaemia. In this context, we have also searched for the visualization of lysophosphatidylserines (LysoPSs), since they appear to be signalling lipids that regulate immunological and neurological processes via membrane receptors, including stimulation of phagocytosis by macrophages^26^,^27^. Furthermore, we have also searched for a number of other signalling lipids, including sphingolipid metabolites, monoacylglycerols (MAGs), and dihydroxy-derivates of docosahexaenoic acid (DHA) and docosapentaenoic acid (DPA). Of the sphingolipid derivates, both sphingosine-1-phosphate (S1P) as an intercellular messenger^28^ and ceramide-1-phosphate (CerP), whether extracellular or intracellular^29^, have been implicated in regulation of neuronal damage^30^,^31^. The endocannabinoid, 2-arachidonoylglycerol (2-AG), is an important neuromodulator in the brain in both normal physiology^32^ as well as during brain injury^33^, but also as a precursor for eicosanoids during neuroinflammation^34^. The tissue level of 2-AG had been reported to be increased at 4 h or at 24 h after permanent damage to the brain (trauma or ischaemia) in mice^13^,^18^,^35^, but it is not known how levels of 2-AG are in the late resolving phase. Within recent years, di- and tri-hydroxylated derivates of DHA and DPA, some of which are called protectins, resolvins, and maresins, have been reported to have several pro-resolving functions in the late stages of inflammation^36^ and possibly also during focal cerebral ischaemia^37^. However, their endogenous time-course of formation in pMCAO is not very clear.

We have used Desorption Electrospray Ionization (DESI) and Matrix Assisted Laser Desorption Ionization (MALDI) imaging in time and space to analyse the involvement of selected lipids in the progression and resolution of the ischaemic insult caused by pMCAO in mice

A list of abbreviations is provided in the supplementary information.

## Results

With DESI imaging of selected phospholipid species, we investigated at different post-surgical survival times the progression of ischaemia. We used permanent MCAO for induction of ischemia, and our results may thus not be applicable for ischemia with transient MCAO, which is a model for the minority of stroke patients having rapid reperfusion treatment at a hospital. For all time points investigated by DESI imaging, we used a spatial resolution of 100 × 100 μm^2^ and measured on sections with 2 h, 24 h, 5d and 20d post-surgical survival. In some of the DESI images, a thin line of increased signal intensity is observed along the edge of the tissue. This “edge effect” is occasionally observed in DESI imaging and may be ascribed to differences in surface charging between the tissue and the bare glass slide rather than actual higher abundances at the edge of the tissue.

### Cessation of the Na^+^/K^+^-ATPase activity and activation of phospholipases

**Figure 1:**
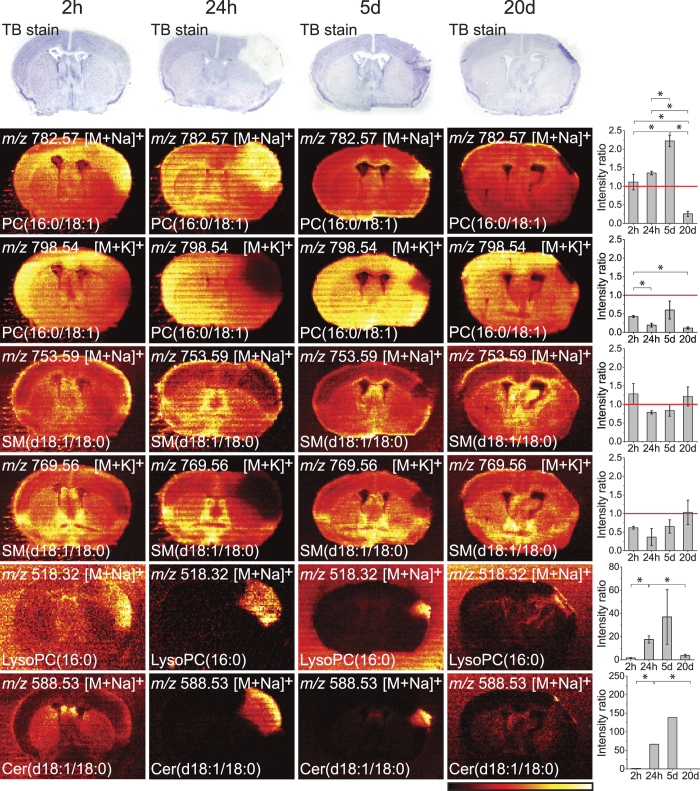
Cessation of Na^+^/K^+^-ATPase activity and activation of lipases. Mouse brains with 2 h, 24 h, 5d and 20d post-surgical survival after the pMCAO-procedure were analysed. The ion images have individual intensity bars between 0–100%, and therefore, the intensity colours cannot be compared between two images. For each lipid, the ratio between the intensity of the ischaemic area and the comparable size area in the contralateral site are shown on the right side where bars are mean ± SEM (n = 3), *p < 0.05. The red line indicates ratio = one. The sodium adduct of PC(16:0/18:1) accumulated in the ischaemic area while the potassium adduct disappeared. This was caused by the cessation of the Na^+^/K^+^-ATPase activity, which indicated that this PC species mainly was found intracellularly. In addition to that, LysoPC(16:0) accumulated because of the activation of one or more different subtypes of phospholipase A_2_. Both the sodium and potassium adducts of SM(d18:1/18:0) disappeared from the area probably caused by sphingomyelinase degrading SM to Cer. Cer(d18:1/18:0) accumulated in the ischaemic area at 24 h and 5d. Since the sodium adduct of SM(d18:1/18:0), unlike the sodium adduct of PC(16:0/18:1), did not increase, we concluded that it was mainly found in the outer leaflet of the plasma membrane. All images were measured in positive ion mode by DESI imaging with a spatial resolution of 100 × 100 μm^2^ and the images are a typical representative of 3 mice. Molecular structures of the lipids can be found in Supplementary Figures S1 and S3.

In positive ion mode, we mainly observed ionized phosphatidylcholine (PC) and to a lesser extent sphingomyelin (SM). PC is found both in the outer leaflet of the plasma membrane and the inner membranes (both leaflets) of the cell, while SM primarily is found in the outer leaflet of the plasma membrane. Thus, those PC species that predominantly are in the intracellular membranes mainly experience a high potassium concentration, while SM that predominantly is in the outer leaflet of the plasma membrane is in contact with a high extracellular sodium concentration. In the ischaemic area, lack of oxygen causes fall in the intracellular ATP concentration, influx of sodium and calcium over the plasma membrane, followed by activation of one or more of the different subtypes of phospholipase A_2_ and sphingomyelinase, which generates lysophosphatidylcholines (LysoPC) and ceramides (Cer) from PC and SM respectively. In Fig. 1, the distribution of the most abundant PC and SM species in the brain, PC(16:0/18:1) and SM(d18:1/18:0), are shown using DESI imaging (for molecular structure and MS/MS spectra for the two lipids, see Supplementary Fig. S1). The sodium adduct of PC(16:0/18:1) accumulated in the ischaemic area at 2 h, 24 h and 5d while it was not observable at 20d in the small remaining injured area. On the other hand the potassium adduct disappeared within the same time frame. This can be explained by the cessation of the Na^+^/K^+^-ATPase activity, as previously reported^13^,^14^. When the pump breaks down sodium floats into the cell causing accumulation of the sodium adduct. Furthermore, the activated phospholipase A_2_ hydrolyses PC(16:0/18:1) into LysoPC(16:0), which accumulates here as the sodium adduct. Contrary to this, both the sodium and potassium adducts of SM(d18:1/18:0) disappeared from the ischaemic area. This may be due to an activated sphingomyelinase, which removes the phosphocholine head group from SM(d18:1/18:0) and generates Cer(d18:1/18:0), here seen as the sodium adduct. This accumulation of Cer(d18:1/18:0) can be observed at 24 h and 5d. The potassium adducts of LysoPC(16:0) and Cer(d18:1/18:0) showed the same trend as the sodium adducts, see supplementary Fig. S2 (for molecular structures for LysoPC(16:0) and Cer(d18:1/18:0) and MS/MS spectra for LysoPC(16:0), see supplementary Fig. S3). The changes in abundance of the sodium and potassium adducts of PC(16:0/18:1) and SM(d18:1/18:0) may also give information about the localizations of these lipids. PC(16:0/18:1) was affected by the cessation of the Na^+^/K^+^-ATPase activity, suggesting that it mainly is localized in the inner leaflet of the plasma membrane as well as the inner membranes of the cell. On the other hand, the sodium adduct of SM(d18:1/18:0) was not accumulated, in accordance with its main localization in the outer leaflet of the plasma membrane and perhaps also caused by breakdown by sphingomyelinase. To make sure that the accumulation of the sodium adduct and the disappearance of the potassium adduct of PC was not caused by ion suppression, we sprayed a 24 h section with PC(10:0/10:0) (not present naturally in the brain) before measuring by DESI imaging. In supplementary Fig. S4 we tested the effect of ion suppression in the ischaemic area compared to healthy brain tissue and concluded that even though the ion suppression of the lipids in the ischaemic area seemed less than in the healthy part, this smaller ion suppression may not explain the observed accumulation of e.g. LysoPC(16:0) and Cer(d18:1/18:0).

### Arachidonic- and docosahexaenoic-rich PCs

Supplementary Fig. S5 shows DESI imaging of the behaviour of the sodium and potassium adducts of the PCs containing arachidonic (AA, 20:4(n-6)) or docosahexaenoic acid (DHA, 22:6(n-3)). As with PC(16:0/18:1), PC(18:0/22:6) is affected by the cessation of the Na^+^/K^+^-ATPase activity. We observed accumulation of the sodium adduct and disappearance of the potassium adduct in the ischaemic area. Accumulation of the sodium and potassium adduct of LysoPC(18:0) could also be seen having the same pattern as the sodium and potassium adduct of LysoPC(16:0), as shown in Supplementary Fig. S7. Accumulation of PC(18:0/20:4) followed the same pattern although with a weaker accumulation of the sodium adduct. However, we did not observe accumulation of the potassium adduct at 24 h and 5d as reported by Hanada *et al*.^12^ in ischaemic spinal cord.

### Accumulation of monoacylglycerols (MAGs) at the resolution stages of ischaemia

**Figure 2:**
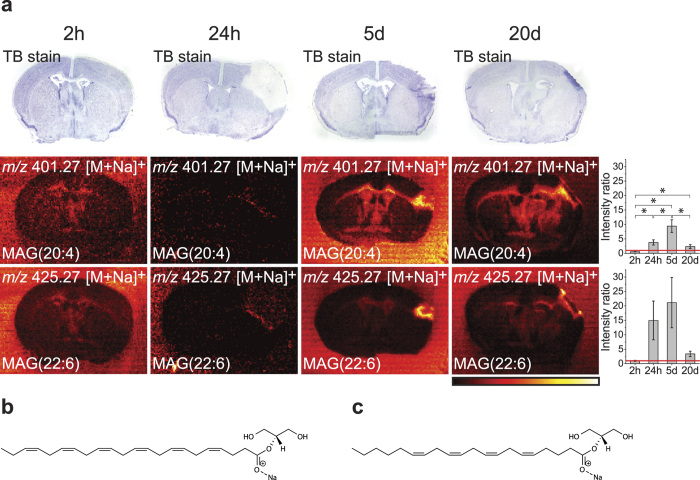
Accumulation of monoacylglycerols. **(a)** MAG(20:4) and MAG(22:6) were measured at 2 h, 24 h, 5d, and 20d. The ion images have individual intensity bars between 0–100%, and therefore, the intensity colours cannot be compared between two images. For each lipid, the ratio between the intensity of the ischaemic area and the comparable size area in the contralateral site are shown on the right side where bars are mean ± SEM (n = 3), *p < 0.05. The red line indicates ratio = one. At 2 h, no accumulation of the lipids could be seen, at 24 h a weak accumulation was seen, and at 5d and 20d a clearer accumulation could be seen in the ischaemic area. All images were measured in positive ion mode using DESI imaging with a spatial resolution of 100  ×  100 μm^2^ and the images are a typical representative of 3 mice. **(b)** Molecular structure MAG(20:4) (here shown as the 2-arachidonoylglycerol sodium adduct ion). **(c)** Molecular structure of MAG(22:6) (here shown as the 2-docosahexaenoylglycerol sodium adduct ion).

Figure 2a shows the tentatively identified distribution of the endocannabinoid MAG(20:4) at the four time points along with MAG(22:6), the suggested molecular structures can be seen in Fig. 2b,c, respectively. At 2 h no accumulation of the two MAGs was observed, and at 24 h a weak accumulation could be seen. At 5d and 20d, accumulation was observed in and especially around the edges of the injured area, where tissue repair is taking place.

### Accumulation of *N*-acyl-phosphatidylethanolamine (NAPE) and bis(monoacylglycero)phosphate (BMP) species seen by DESI imaging

**Figure 3:**
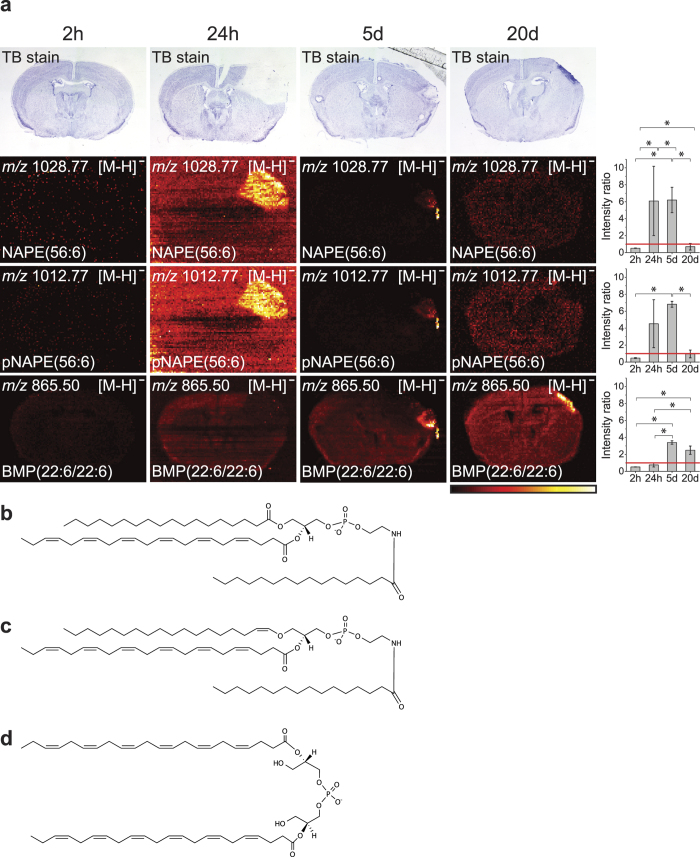
Accumulation of BMP and NAPE over time during ischaemia. **(a)** The distribution of NAPE(56:6), pNAPE(56:6), and BMP(22:6/22:6) were investigated. The ion images have individual intensity bars between 0–100%, and therefore, the intensity colours cannot be compared between two images. For each lipid, the ratio between the intensity of the ischaemic area and the comparable size area in the contralateral site are shown on the right side where bars are mean ± SEM (n = 3), *p < 0.05. The red line indicates ratio = one. The NAPE and pNAPE species accumulated both evenly over the ischaemic area at 24 h. At 5 days, NAPE and pNAPE also accumulated, however, the abundance of the two lipids were not evenly distributed throughout the area. At 20d, NAPE and pNAPE were not detected. BMP was not accumulating until 5d where it seemed to be most abundant at the edge, but was in turn still present in the ischaemic area at 20d, where it was evenly spread throughout the ischaemic area. All images were measured in negative ion mode using DESI imaging with a spatial resolution of 100 × 100 μm^2^ and the images are a typical representative of 3 mice. Molecular structure of **(b)** NAPE(56:6) (here shown as NAPE(18:0/22:6/16:0)), **(c)** pNAPE(56:6) (here shown as pNAPE(18:0/22:6/16:0), and **(d)** BMP(22:6/22:6).

In positive ion mode, the observed lipids were mainly seen as sodium and potassium adducts, but in negative ion mode they were mainly observed as the deprotonated ion. Figure 3a shows the accumulation of NAPE species and BMP(22:6/22:6) during the progression of ischaemia. NAPE(56:6) and pNAPE(56:6) accumulated in the ischaemic area at 24 h and 5d, but no accumulation were observed at 2 h. At 20d, NAPE and pNAPE had again disappeared. There was no BMP(22:6/22:6) present at 2 h and 24 h, but at 5d accumulation of BMP(22:6/22:6) could be seen, especially in the edge of the ischaemic area. At 20d, BMP(22:6/22:6) was spread out to accumulate in the entire region of the remaining injured area. Comparing BMP(22:6/22:6) to NAPE, we saw that the NAPE species were localized all over the ischaemic area at 24 h with no BMP(22:6/22:6) present. At 5d, BMP(22:6/22:6) had begun to accumulate and the NAPE species were no longer present evenly over the ischaemic area, but had lesser abundance in the regions where BMP(22:6/22:6) were located. Going to 20d, the NAPE species had disappeared while BMP(22:6/22:6) had spread to the total remaining injured area. Molecular structures of NAPE(56:6), pNAPE(56:6), and BMP(22:6/22:6) are shown in Fig. 3b–d and MS/MS spectra and DESI imaging of MS/MS fragments can be seen in Supplementary Figs S8, S9 and S10 for NAPE(56:6), pNAPE(56:6), and BMP(22:6/22:6) respectively.

### Accumulation of cholesteryl esters (CE) in the resolution phases of inflammation

Cholesteryl esters (CEs) were seen to accumulate in the ischaemic area during the resolution phases of inflammation (at day 5 and 20). Supplementary Fig. S11a shows the distribution of CE(18:1), CE(20:4), and CE(22:6) (the molecular structures can be seen in Supplementary Fig. S11b, c and d, respectively). At 2 h, no accumulation of CE could be observed, however, at 24 h a weak accumulation of CE(18:1) and CE(20:4) in the ischaemic area were seen. At 5d and 20d a strong accumulation of all three CE species were observed. The localization of CE seemed to follow that of BMP (Fig. 3).

With our MALDI imaging setup, we were able to investigate brain sections with higher spatial resolution (smaller pixel size) and thus were able to investigate the sections in greater spatial detail. We measured on sections with 5d, 7d and 20d post-surgical survival using MALDI imaging.

### Accumulation of NAPE and BMP in the ischaemic area

**Figure 4:**
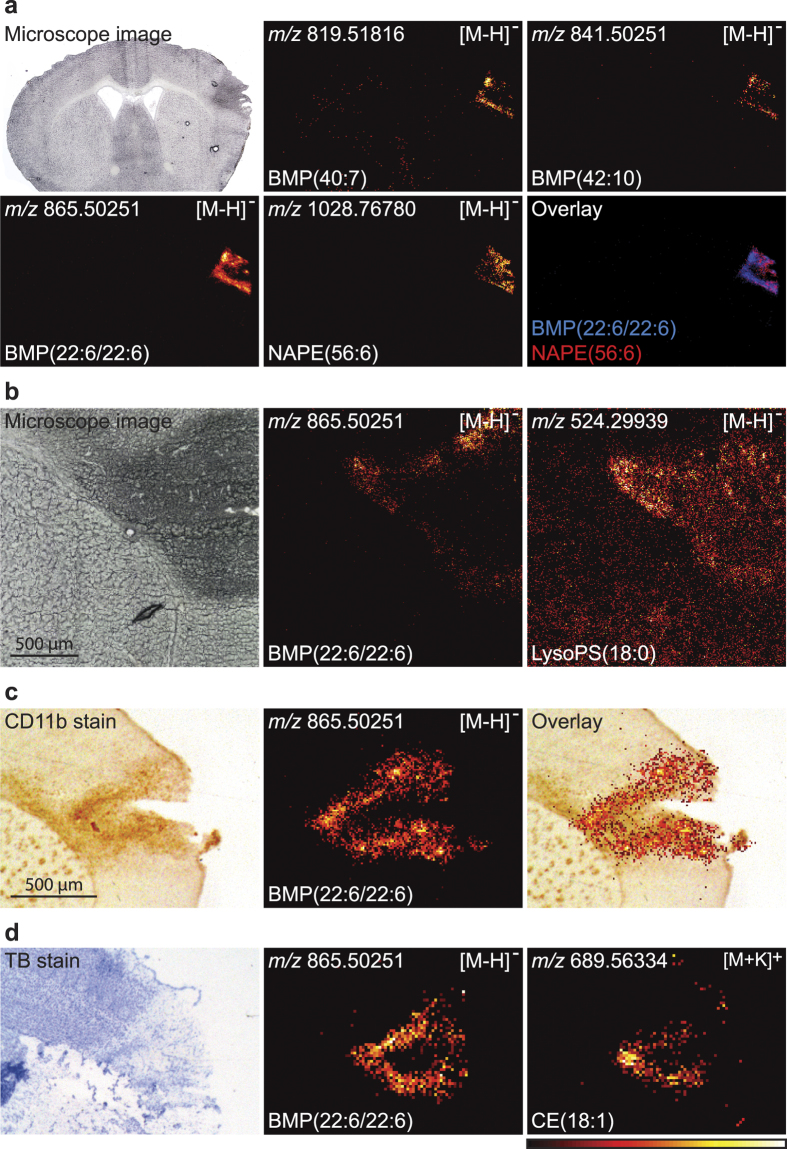
BMP as a biomarker for phagocytosis. MALDI imaging showed the presence of more species of BMP. **(a)** BMP(40:7), BMP(42:10), and BMP(22:6/22:6) all accumulated in the edge of the ischaemic area at day 5 The ion images have individual intensity bars between 0–100%, and therefore, the intensity colours cannot be compared between two images. BMP(22:6/22:6) seemed though to be the most abundant of the three BMP species. The images were measured with a spatial resolution of 35 × 35 μm^2^. **(b)** Looking at BMP(22:6/22:6) with a higher spatial resolution clearly showed that it accumulated at the edge of the ischaemic area. Abundance of LysoPS(18:0) was seen faintly distributed throughout the section as well as a clear accumulation at the edges of the ischaemic area coinciding with BMP(22:6/22:6). The images were measured with a spatial resolution of 7 × 7 μm^2^. **(c)** After measuring the distribution of BMP(22:6/22:6), the tissue was stained for CD11b to compare with the distribution of macrophages/microglia cells. Co-localization of BMP(22:6/22:6) and CD11b is shown by overlay. The image was measured with a spatial resolution of 15 × 15 μm^2^. **(d)** The section was first measured in negative ion mode to visualize the distribution of BMP(22:6/22:6) and then measured in positive ion mode to visualize the distribution of the potassium adduct of CE(18:1). The images were measured with a spatial resolution of 35 × 35 μm^2^. All images were measured by MALDI imaging on sections with 5 day post-surgical survival and the images are a typical representative of 3 mice, except 4d, which is a typical representative of 2 mice. Molecular structures of BMP(40:7), BMP(42:10, and LysoPS(18:0) are shown in Supplementary Fig. S12.

With DESI imaging, we were only able to find one BMP species, BMP(22:6/22:6). However, measuring the samples using MALDI imaging showed us the presence of other BMP species in the ischaemic area. Figure 4a shows that, along with BMP(22:6/22:6), we were able to find species tentatively identified as BMP(40:7) and BMP(42:10) in brains with 5d post-surgical survival (see their molecular structure in Supplementary Fig. S12a and b). Comparing the localization of BMP(22:6/22:6) with NAPE(56:6) (Fig. 4), we could see that the two lipids were mainly localized in two different regions of the ischaemic area suggesting that where macrophages/microglia cells (i.e. BMP as a biomarker for phagocytosis) had phagocytized the dead neurones, NAPE was no longer present (i.e. NAPE as a biomarker for dead/dying neurones). NAPE was still present in the area where BMP had not yet accumulated.

### BMP as a lipid biomarker for macrophages/microglia cells

LysoPS has been identified as a pro-resolving signalling lipid implicated in macrophage activation and clearance of the apoptotic cells^27^. Figure 4b shows the distribution of BMP(22:6/22:6) and LysoPS(18:0) in the ischaemic area in a sample with 5d post-surgical survival. While LysoPS(18:0) is distributed faintly throughout the section, accumulation of LysoPS could also clearly be observed in the edges of the ischaemic area, coinciding with the distribution of BMP(22:6/22:6) (See the molecular structure of LysoPS(18:0) in Supplementary Fig. S12c).

To compare the localization of macrophages/microglia cells with BMP, we performed immunohistochemistry on the sections by staining for CD11b, a biomarker for macrophages/microglia cells^38^. In Fig. 4c, we visualized a brain section of 5d post-surgical survival using MALDI imaging, followed by immunohistochemical staining of the section for CD11b. The distribution of CD11b and BMP(22:6/22:6) coincide, supporting our claim that BMP can be used as a biomarker for phagocytizing macrophages/microglia cells. Higher spatial resolutions pictures of CD11b clearly showing the staining of individual microglia/macrophages are shown in Supplementary Fig. S13. Furthermore, in Fig. 4d we compared the spatial distribution of BMP(22:6/22:6) with the potassium adduct of CE(18:1) on a mouse brain with 5d post-surgical survival by measuring the tissue in both negative and positive ion mode. Here we found that BMP and CE co-localized.

Since BMP is especially abundant in alveolar macrophages^24^, we analysed BMP in TiO_2_-nanoparticle-exposed mouse lungs and in normal control lungs. These MSI images clearly show that BMP(22:6/22:6), and LysoPS(18:0) were increased in the TiO_2_- nanoparticle-exposed lungs compared to the control lungs (see Supplementary Fig. S14).

### Sphingosine-1-phosphates accumulate in the resolution phase of inflammation

**Figure 5:**
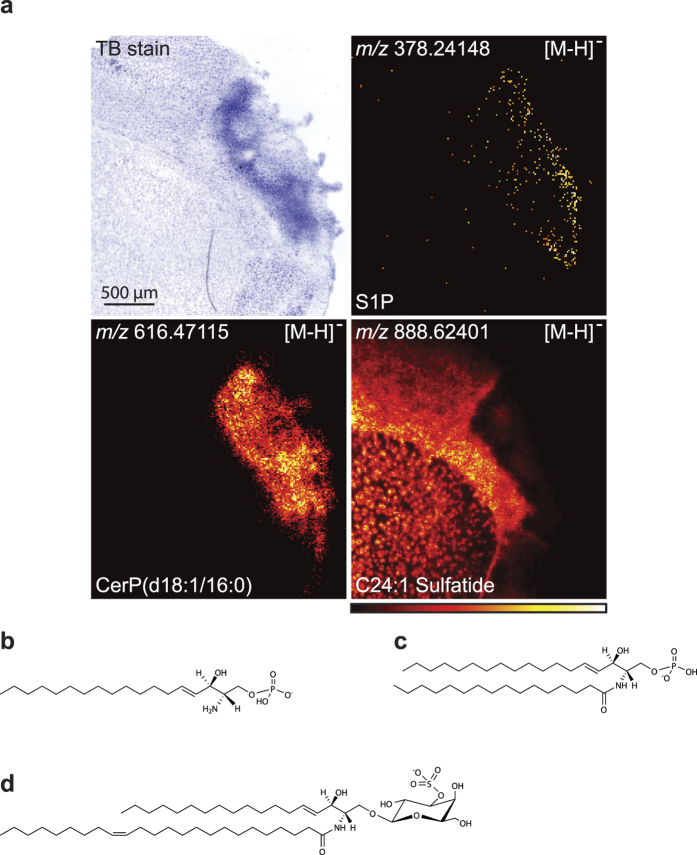
Accumulation of Sphingosine-1-phosphate and CerP species. **(a)** S1P and CerP(d18:1/16:0) were found to accumulate in the ischaemic area at 7d post-surgical survival. In contrast, C24:1 Sulfatide disappeared from the area. All images were measured in negative ion mode using MALDI imaging with a resolution of 15 × 15 μm^2^ and the images are a typical representative of 3 mice. Molecular structures of **(b)** S1P, **(c)** CerP(d18:1/16:0), and **(d)** C24:1 Sulfatide.

Sphingosine-1-phosphate has emerged as an important mediator in inflammation regulating immune cell trafficking^39^. Figure 5a shows the accumulation of sphingosine-1-phosphate and CerP(d18:1/16:0) along with the disappearance of C24:1 sulfatide in the ischaemic area in a mouse brain with 7d post-surgical survival. The molecular structures of S1P, CerP(d18:1/16:0), and C24:1 Sulfatide are shown in Fig. 5b to d.

### Localization of *N*-acyl-taurines (NAT) in the resolution phases of inflammation

**Figure 6:**
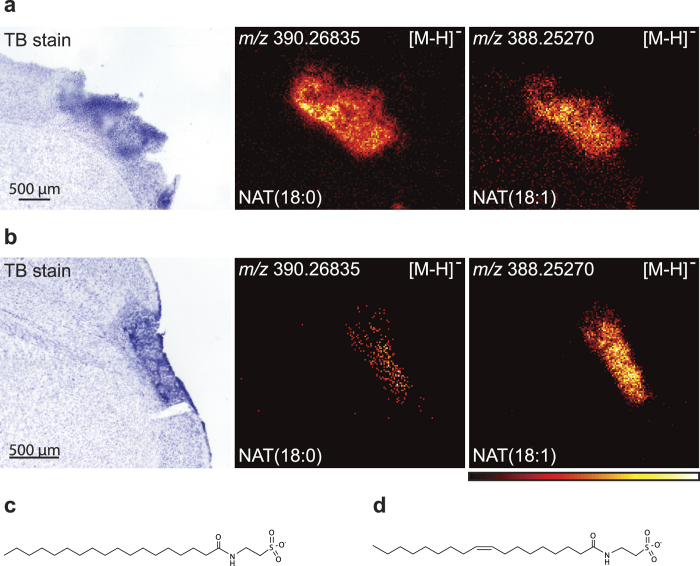
*N*-acyl-taurines accumulate in the resolution phase of inflammation. NAT(18:0) and NAT(18:1) were both found to accumulate in the ischaemic area at **(a)** 7 days and **(b)** 20 days. The images were measured at 7d and 20d in negative ion mode using MALDI imaging with a resolution of 25 × 25 μm^2^ and 15 × 15 μm^2^ respectively. The images are a typical representative of 3 mice. Molecular structures of **(c)** NAT(18:0) and **(d)** NAT(18:1).

Unexpectedly, we found that several species of *N*-acyl-taurine (NAT) were accumulating in the ischaemic area in the resolution phase, while they were not visible at the earlier time points. Figure 6a,b shows the distribution of NAT(18:0) and NAT(18:1) at 7d and 20d, respectively. Their molecular structures are shown in Fig. 6c,d.

### Fatty acids and their derivates in the resolution phases of inflammation

**Figure 7:**
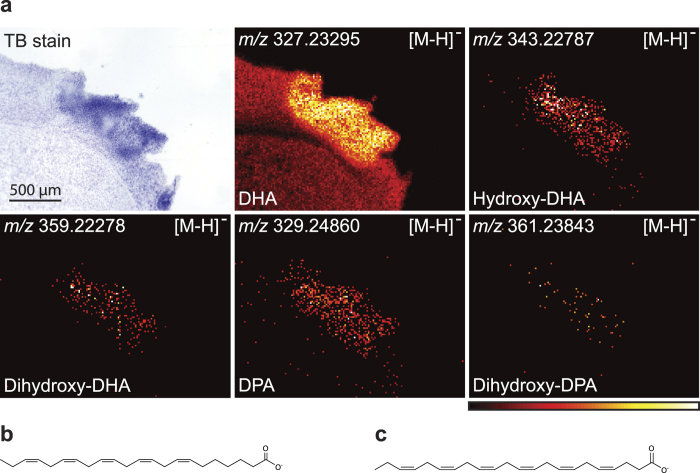
DHA and DPA are precursors for lipid mediators, which play a role in the resolution phase of inflammation. **(a)** DHA and DPA were accumulating in the ischaemic area at 7d post-surgical survival. Likewise, hydroxy-derivatives of these fatty acids were found to accumulate: hydroxy-DHA, dihydroxy-DHA, and dihydroxy-DPA were all accumulating in the ischaemic area. These images were measured at 7d in negative ion mode using MALDI imaging with a resolution of 15 × 15 μm^2^ and the images are a typical representative of 3 mice. Molecular structures of **(b)** DHA and **(c)** DPA.

The importance of oxygenated derivatives of liberated fatty acids in the resolution of ischaemia has been realized in recent years. Important fatty acid precursors include DPA (22:5(n-3)) and DHA (22:6(n-3))^36^. In Fig. 7a, we show the accumulation of DHA, hydroxy-DHA, dihydroxy-DHA, DPA, and dihydroxy-DPA in the ischaemic area after 7d post-surgical survival. While these derivates were observed at 7 days, we were not able to find them in any of our sections with other survival times (2 h, 24 h, 5d and 20d). Due to their low abundance, we could not perform stereo-chemical identification revealing whether they were resolvins, maresins, protectins or other derivatives. The molecular structures of DHA and DPA are shown in Fig. 7b,c.

## Discussion

The application of MSI for the study of spatiotemporal changes of the inflammatory lipidome during focal cerebral ischaemia has brought a number of significant biological results^11^,^13^. Initially, we have studied the lipidome using the DESI imaging setup, while we later also has access to the MALDI imaging setup, which provide an accurate mass facilitating molecular identification. First, we provide evidence that BMP can be used as a biomarker for phagocytizing macrophages/microglia cells in the late resolving state of inflammation as BMP co-localized with the macrophage/microglia cells biomarker CD11b. Furthermore, our supplementary studies of abundance of BMP in alveolar macrophages support this conclusion. Several different species of BMP could be visualized with BMP(22:6/22:6) being the most abundant in the brain, while BMP(36:2) was the most abundant in the lung. Furthermore, in the same brain section high abundance of LysoPS(18:0) was also observed. LysoPS is a bioactive lipid mediator, which via activation of G-protein-coupled receptors seems to be involved in stimulation of phagocytosis by macrophages during resolution of inflammation^27^. It was only at day 5 we were able to observe LysoPS in the infarcted area, and not at an earlier time-point. In contrast, LysoPC(16:0) was very abundant at 2 h and 24 h, although it was still present at 5d and 20d. This seems to be in agreement with the generation of LysoPC from injured astrocytes and neurones and that LysoPC may then stimulate the activation of microglia cells^40^. We were not able to find lipid biomarkers, which specifically could visualize the existence of a penumbra within the first 2 hours. NAPE species slowly accumulate during cell death^41^,^42^,^43^ especially in neurones as opposed to astrocytes^22^,^41^,^43^, and we argue that the increased abundance of NAPE in the ischaemic area is caused primarily by death of the neurones, which eventually disappear due to phagocytosis by invading macrophages/microglia cells. Cholesteryl esters accumulated in the late resolving phase, as also seen by Roux *et al*.^44^. We found that CE(18:1) to some extent co-localized with BMP(22:6/22:6) suggesting that cholesteryl esters accumulate in the macrophages/microglia cells due to phagocytosis of cholesterol-containing dead cells/cell debris. Using higher spatial resolution with MALDI imaging, we could see that NAPE did not co-localize with BMP(22:6/22:6) suggesting that where NAPE is localized, dead neurones are still present, and where BMP is located dead neurones have been degraded by phagocytosis by macrophages. As previously reported^13^,^14^, a clear change in the ratio of the potassium and sodium adducts of several PC species (e.g. PC(16:0/18:1) and PC(18:0/22:6)) was seen as an indication of their intracellular localization and a cessation of the Na^+^/K^+^-ATPase activity due to lack of ATP. However, the sodium adduct of PC(18:0/20:4) did not clearly increase while the potassium adduct did decrease. Whether this is due to this lipid serving as a precursor for generation of LysoPC and arachidonic acid (AA, 20:4(n-6)) metabolites is not clear. M. Hanada *et al*.^12^ observed that AA-rich PC was temporally elevated one week after spinal cord injury and interpreted this as caused by invasive immune cells. We did not observe such a temporally increase in AA-rich PC species. The abundance in the ischaemic area of sodium and potassium adducts of SM(d18:1/18:0) decreased both in the early phase and the late phase. This may be due to the localization of sphingomyelin mainly in the extracellular leaflet of the plasma membrane as well as to the concomitant generation of ceramide (seen both as sodium and potassium adducts of Cer(d18:1/18:0)) from sphingomyelin. This generation of ceramide during cell death of ischaemia is well-established^45^ also in MSI studies^14^,^15^,^46^. However, we also observed an increased abundance of both ceramide-1-phosphate (CerP(d18:1/16:0)) and sphingosine-1-phosphate (S1P) in the ischaemic area at day 7, with CerP(18:1/16:0) having high abundance in the whole ischaemic area whereas S1P was faintly seen in the periphery of the ischaemic area. Both of these lipids have signalling functions^28^,^47^ and their formation in the later phase of inflammation may suggest that they have some important functions related to the resolution of inflammation. S1P has been found to have an inhibitory function on vascular inflammation^48^.

The endocannabinoid 2-arachidonoylglycerol (2-AG) has been implicated as a neuroprotective factor generated during the early phase of ischaemia^35^,^49^,^50^. In the present study, we especially saw increased abundance of MAG(20:4) also in the later resolution phase of ischaemic inflammation (5d and 20d) while at the same time MAG(22:6) also increased in abundance. Since, 2-AG is much more abundant in the brain than 1-AG^51^, we assume that our MAG(20:4) is mainly 2-AG and that MAG(22:6) is mainly 2-docosahexaenoylglycerol (2-DHA-G) respectively. It is not clear whether 2-AG served as agonist for cannabinoid receptor-1 and cannabinoid receptor-2^33^ or whether it primarily served as a precursor molecule for formation of various eicosanoids^34^,^52^. Lipoxin A4 generated from arachidonic acid is known as a pro-resolving lipid mediator^53^, but a number of dihydroxy-derivates of both docosahexaenoic acid (DHA) and docosapentaenoic acid (DPA) have also various pro-resolving activities, having names as resolvins, maresins and protectins^36^. We observed localization of dihydroxy-DHA and dihydroxy-DPA as well as their precursors DHA and DPA in the ischaemic area at day 7, identified by their exact mass, within the ischaemic area. It was not possible to identify the exact chemical structures of these di-hydroxy fatty acids and thereby more exactly suggest their possible biological functions. Generally, it is well-known that lack of oxygen leads to post-mortem changes in levels of fatty acids and other signalling lipids^54^ as well as of several small water-soluble metabolites^55^,^56^. In Fig. 7, DHA is seen in both the ischaemic area and in the non-ischaemic area. Furthermore, the decapitation procedure can cause artificial alterations in metabolic profiles in some metabolic pathways in the apparently healthy contralateral hemisphere, e.g. increase of adenosine monophosphate^57^.

We believe that the reason for seeing some DHA in the non-ischaemic area is due to post-mortem accumulation during sampling of the brains^54^. However, DHA intensity is clearly higher in the infarct area reflecting a specific release of DHA in this area. By studying the lipids in a defined area with tissue infarct, the non-infarct tissue can serve as a sort of control for infarct-specific changes in levels of the lipids, i.e. changes not caused by post-mortem lack of oxygen. We also found that many species of *N*-acyl-taurines (NATs) accumulated in the injured area in the resolving phase (7d and 20d). Not much is known about biological functions of NATs, but it has been reported that they can activate TRP receptor^58^ and inhibit proliferation of prostate cancer cells^59^. Our finding of high abundance of NATs during resolution of inflammation raises the question whether they have anti-inflammatory functions.

In conclusion, our MSI study has shown that (A) BMP can be used as a biomarker of phagocytizing macrophages/microglia cells in histological studies, (B) NAPE may be a marker for dying/dead neurones, C) the ratio of Na^+^/K^+^-adducts of selected choline-containing phospholipids in dying cells can suggest whether the lipids are localized intracellularly or on the outer leaflet of the plasma membrane, and D) a number of both pro-inflammatory and pro-resolving lipid mediators change in abundance between the early pro-inflammatory and the late pro-resolving phases of neuroinflammation. Furthermore, this lipidome technique may discover new lipid species involved in the inflammatory process. With the present MSI lipidome techniques combined with immunohistochemistry, it will in the future be possible to dissect in greater detail the spatiotemporal changes of cells and of lipid and peptide mediators during an inflammatory process, and suggests more precise biological roles for the various cell types and mediator compounds involved.

## Methods

### Induction of brain ischaemia

Focal cerebral ischaemia was induced in anaesthetized 7- to 8-week-old C57BL/6 male mice by permanent middle cerebral artery occlusion (pMCAO) of the distal part of the left middle cerebral artery, as previously described^38^. Mice were obtained from The Jackson Laboratory (Maine, USA) and were cared for in accordance with the protocols and guidelines approved by the Danish Animal Inspectorate (J number 2013-15-2934-00924). All efforts were made to minimize pain and distress.

### Tissue preparation

Mice were decapitated after cervical dislocation at a range of post-surgical survival times. Survival times selected for DESI imaging were 2 h, 24 h, 5d, and 20d, and for MALDI imaging 5d, 7d, and 20d with three mice at each survival time. The brains were quickly removed from the skulls, frozen in gaseous CO_2_, and subsequently cut into 30 μm thick coronal cryostat sections. Sections were placed on microscope slides and stored in sealed boxes at −80 °C. Before MSI a section was removed from the freezer and placed in a vacuum desiccator for approximately 10 min. to remove water and thus prevent enzymatic reactions in the brain tissue during the measurement.

### Desorption electrospray ionization (DESI) imaging

DESI imaging was performed on a LTQ XL linear ion trap mass spectrometer (Thermo Scientific, California, USA) equipped with a custom-built DESI imaging ion source, as previously described^60^. The electrospray was constructed of coaxial fused silica capillaries connected in 1/16-inch Swagelok tee (Swagelok Co., USA), an inner capillary (50 μm ID, 150 μm OD, SGE, USA) carrying the spray solvent, and an outer capillary (250 μm ID, 350 μm OD, SGE, USA) carrying the nebulizer gas. The electrospray was directed toward the surface of the sample in order to desorb and ionize compounds on the surface followed by analysis in the mass spectrometer.

The solvent spray consisted of methanol and water (95:5) dispensed with a flow of 5 μl/min and the nitrogen nebulizer gas was set to a pressure of 9 bar. Each mass spectrum was measured with an injection time of 100 ms and an average of 5 microscans for positive ion mode and an injection time of 200 ms with an average of 3 microscans for negative ion mode. The spray-to-inlet and spray-to-sample distance were optimized to approximately 4.5 mm and 1.5 mm respectively with a spray angle of approximately 55°. The spray potential was 5 kV for positive ion mode and −5 kV for negative ion mode, and the mass-to-charge (*m/z*) scan range was set between 250 and 1100 for all measurements.

Placed on a moving stage, the section was moved under the electrospray for 100 μm during measurement of one mass spectrum. The whole section was recorded line-by-line with a distance of 100 μm between each line giving a spatial resolution of 100 × 100 μm^2^. In both positive and negative ion mode, measurements were performed on brain sections from three different mouse brains, n = 3, and a typical representative were used for the images in the figures.

### Matrix assisted laser desorption ionization (MALDI) imaging

A solution of 150 μl 4-nitroaniline (10 mg/mL in acetone/water (50:50, v/v)) was sprayed on the sections with a pneumatic sprayer, as described by Bouschen, W. *et al*.^61^, with a flow of 10 μl/min and a pressure of 1 to 2 bar nitrogen gas. While being sprayed the section was rotated with approximately 300 to 500 rpm.

After matrix application the sample was placed in an atmospheric-pressure scanning-microprobe matrix assisted laser desorption/ionization imaging source (AP-SMALDI10, TransMIT GmbH, Giessen, Germany) coupled to a Fourier transform orbital trapping mass spectrometer (QExactive, Thermo Fisher Scientific GmbH, Bremen, Germany). For analyte ionization, a nitrogen laser with a wavelength of 337 nm and a frequency of 60 Hz with 30 pulses per shot was used. The laser beam spot size was focused on the sections to match the resolution, between 7 × 7 and 50 × 50 μm^2^, for a given measurement. Sections were measured in negative ion mode with different *m/z* ranges between 250 and 1200 or positive ion mode with *m/z* range 250 to 1000. The mass resolution was 70,000 or 140,000 with the automatic gain control turned off and with a fixed injection time of 500 ms (microscans = 1) to match the time of one mass spectrum with the time of one 30 laser pulses.

The measurements in negative ion mode were performed on brain sections from three different mouse brains, n = 3, and a typical representative were used for the images in the figures. The only exception to this were the images shown in Fig. 4e where tissue sections were measured first in negative and then in positive ion mode to compare BMP with CE. These were only measured on tissue sections from 2 different mouse brains.

### Microscope images/Toluidine Blue stains

Sections measured in Copenhagen, Denmark were after the measurement stained with 0.5% Toluidine Blue (Fluka Analytical, Sigma-Aldrich, Missouri, USA) in water for approximately 8 min., and then dehydrated in graded series of alcohol (70–99%), cleared in xylene, and finally cover-slipped with Eukitt quick-hardening mounting medium (Fluka Analytical, Sigma-Aldrich, Missouri, USA). The Toluidine Blue (TB) stains were then captured on a Stemi DV4 Stereoscope (Carl Zeiss AG, Oberkochen, Germany) equipped with an LCMOS digital streaming camera (Brunel Microscopes Ltd, Chippenham, UK). An Olympus BX-41 (Olympus Europa, Hamburg, Germany) microscope was used to make optical images of samples measured in Giessen, Germany prior to MALDI imaging. Images composed of more than one image were stitched by the Image Composite Editor (Microsoft Corporation, Washington, USA).

### Data analysis of DESI and MALDI imaging

The Raw-files were converted to imzML by an imzML-converter^62^ and loaded into the open-source MS imaging software, MSiReader^63^. Images were generated for the *m/z* values of interest with a bin width of ±0.1 Da for DESI imaging and ±5 ppm (±0.002 Da to ±0.004 Da) for MALDI imaging. The images shown in the figures are representatives of images from the three different mouse brains measured at each survival time for both DESI and MALDI imaging. To give the best presentation of the images, both on screen and on print, the MATLAB (MathWorks, Massachusetts, USA) colormap ‘Hot’ was chosen. In some of the figures, semi-quantitative data are given for changes in the abundance of the lipids. This has been done by dividing the mean intensity in the injured area with the mean intensity in a comparable area on the contralateral site of the brain section. This was, however, not possible in Fig. 4a due to zero intensity on the contralateral tissue. The data are presented as intensity ratio, mean ± SEM, n = 3 animals. Statistical analysis was performed using one-way ANOVA or, if the intensity ratios did not have a normal distribution, ANOVA on ranks and statistical significance (*p < 0.05) was determined by using the Student-Newman-Keuls Method. Note that the ischaemic area did vary greatly at the different survival times with 24 h having the largest area and 20d the smallest.

### Immunohistochemistry for CD11b

Immunohistochemical staining for CD11b (macrophages/microglia cells) was done with a horseradish peroxidase technique as previously described^38^. The 4-nitroaniline-matrix was removed from the sections by flushing ethanol on the section until the matrix was washed of (around 15 s) and it was then left to dry before beginning of the staining procedure.

### Designing figures

The figures were composed by loading the generated images from MSiReader together with the corresponding microscope image or TB stain into Adobe Illustrator (Adobe Systems, California, USA). Arial was used as font for all figures. DESI images were cropped to minimize the surrounding border of the tissue in Adobe Photoshop (Adobe Systems, California, USA) before being loaded into Adobe Illustrator.

## Additional Information

**How to cite this article**: Nielsen, M. M. B. *et al*. Mass spectrometry imaging of biomarker lipids for phagocytosis and signalling during focal cerebral ischaemia. *Sci. Rep.* **6**, 39571; doi: 10.1038/srep39571 (2016).

**Publisher's note:** Springer Nature remains neutral with regard to jurisdictional claims in published maps and institutional affiliations.

## Supplementary Material

Supplementary Information

## References

[b1] Berry K. A. . MALDI imaging of lipid biochemistry in tissues by mass spectrometry. Chemical reviews 111, 6491–6512, doi: 10.1021/cr200280p (2011). 21942646 PMC3199966

[b2] Goto-Inoue N., Hayasaka T., Zaima N. & Setou M. Imaging mass spectrometry for lipidomics. Biochimica et biophysica acta 1811, 961–969, doi: 10.1016/j.bbalip.2011.03.004 (2011). 21440085

[b3] Sparvero L. J. . Mass-spectrometry based oxidative lipidomics and lipid imaging: applications in traumatic brain injury. Journal of neurochemistry 115, 1322–1336, doi: 10.1111/j.1471-4159.2010.07055.x (2010). 20950335 PMC3285274

[b4] Hanrieder J., Phan N. T., Kurczy M. E. & Ewing A. G. Imaging mass spectrometry in neuroscience. ACS chemical neuroscience 4, 666–679, doi: 10.1021/cn400053c (2013). 23530951 PMC3656743

[b5] Gallala H. D. & Sandhoff K. Biological function of the cellular lipid BMP-BMP as a key activator for cholesterol sorting and membrane digestion. Neurochemical research 36, 1594–1600, doi: 10.1007/s11064-010-0337-6 (2011). 21136156

[b6] Wellner N., Diep T. A., Janfelt C. & Hansen H. S. N-acylation of phosphatidylethanolamine and its biological functions in mammals. Biochimica et biophysica acta 1831, 652–662, doi: 10.1016/j.bbalip.2012.08.019 (2013). 23000428

[b7] Lambertsen K. L., Biber K. & Finsen B. Inflammatory cytokines in experimental and human stroke. Journal of cerebral blood flow and metabolism: official journal of the International Society of Cerebral Blood Flow and Metabolism 32, 1677–1698, doi: 10.1038/jcbfm.2012.88 (2012). PMC3434626 22739623

[b8] del Zoppo G. J., Sharp F. R., Heiss W. D. & Albers G. W. Heterogeneity in the penumbra. Journal of cerebral blood flow and metabolism: official journal of the International Society of Cerebral Blood Flow and Metabolism 31, 1836–1851, doi: 10.1038/jcbfm.2011.93 (2011). PMC3185890 21731034

[b9] Serhan C. N. Pro-resolving lipid mediators are leads for resolution physiology. Nature 510, 92–101, doi: 10.1038/nature13479 (2014). 24899309 PMC4263681

[b10] Gronberg N. V., Johansen F. F., Kristiansen U. & Hasseldam, Leukocyte infiltration in experimental stroke. Journal of neuroinflammation 10, 115, doi: 10.1186/1742-2094-10-115 (2013). 24047275 PMC3852747

[b11] Koizumi S. . Imaging mass spectrometry revealed the production of lyso-phosphatidylcholine in the injured ischemic rat brain. Neuroscience 168, 219–225, doi: 10.1016/j.neuroscience.2010.03.056 (2010). 20362643

[b12] Hanada M. . Spatiotemporal alteration of phospholipids and prostaglandins in a rat model of spinal cord injury. Analytical and bioanalytical chemistry 403, 1873–1884, doi: 10.1007/s00216-012-5900-3 (2012). 22415026

[b13] Janfelt C. . Visualization by mass spectrometry of 2-dimensional changes in rat brain lipids, including N-acylphosphatidylethanolamines, during neonatal brain ischemia. FASEB journal: official publication of the Federation of American Societies for Experimental Biology 26, 2667–2673, doi: 10.1096/fj.11-201152 (2012). 22389441

[b14] Hankin J. A. . MALDI mass spectrometric imaging of lipids in rat brain injury models. Journal of the American Society for Mass Spectrometry 22, 1014–1021, doi: 10.1007/s13361-011-0122-z (2011). 21953042 PMC4537074

[b15] Wang H. Y., Wu H. W., Tsai P. J. & Liu C. B. MALDI-mass spectrometry imaging of desalted rat brain sections reveals ischemia-mediated changes of lipids. Analytical and bioanalytical chemistry 404, 113–124, doi: 10.1007/s00216-012-6077-5 (2012). 22610601

[b16] Girod M., Shi Y., Cheng J.-X. & Cooks R. G. Mapping Lipid Alterations in Traumatically Injured Rat Spinal Cord by Desorption Electrospray Ionization Imaging Mass Spectrometry. Analytical chemistry 83, 207–215, doi: 10.1021/ac102264z (2011). 21142140 PMC3013616

[b17] van Meer G. Lipid traffic in animal cells. Annual review of cell biology 5, 247–275, doi: 10.1146/annurev.cb.05.110189.001335 (1989). 2688705

[b18] Degn M. . Changes in brain levels of N-acylethanolamines and 2-arachidonoylglycerol in focal cerebral ischemia in mice. Journal of neurochemistry 103, 1907–1916, doi: 10.1111/j.1471-4159.2007.04892.x (2007). 17868306

[b19] Hansen H. H., Ikonomidou C., Bittigau P., Hansen S. H. & Hansen H. S. Accumulation of the anandamide precursor and other N-acylethanolamine phospholipids in infant rat models of *in vivo* necrotic and apoptotic neuronal death. Journal of neurochemistry 76, 39–46 (2001). 11145976 10.1046/j.1471-4159.2001.00006.x

[b20] Hansen H. H. . Anandamide, but not 2-arachidonoylglycerol, accumulates during *in vivo* neurodegeneration. Journal of neurochemistry 78, 1415–1427 (2001). 11579150 10.1046/j.1471-4159.2001.00542.x

[b21] Di Marzo V. . Formation and inactivation of endogenous cannabinoid anandamide in central neurons. Nature 372, 686–691, doi: 10.1038/372686a0 (1994). 7990962

[b22] Hansen H. S. . Characterization of glutamate-induced formation of N-acylphosphatidylethanolamine and N-acylethanolamine in cultured neocortical neurons. Journal of neurochemistry 69, 753–761 (1997). 9231736 10.1046/j.1471-4159.1997.69020753.x

[b23] Scherer M. & Schmitz G. Metabolism, function and mass spectrometric analysis of bis(monoacylglycero)phosphate and cardiolipin. Chemistry and physics of lipids 164, 556–562, doi: 10.1016/j.chemphyslip.2011.06.007 (2011). 21704024

[b24] Akgoc Z., Iosim S. & Seyfried T. N. Bis(monoacylglycero)phosphate as a Macrophage Enriched Phospholipid. Lipids 50, 907–912, doi: 10.1007/s11745-015-4045-5 (2015). 26205346

[b25] Desjardins M. . Molecular characterization of phagosomes. The Journal of biological chemistry 269, 32194–32200 (1994). 7798218

[b26] Kamat S. S. . Immunomodulatory lysophosphatidylserines are regulated by ABHD16A and ABHD12 interplay. Nature chemical biology 11, 164–171, doi: 10.1038/nchembio.1721 (2015). 25580854 PMC4301979

[b27] Frasch S. C. & Bratton D. L. Emerging roles for lysophosphatidylserine in resolution of inflammation. Progress in lipid research 51, 199–207, doi: 10.1016/j.plipres.2012.03.001 (2012). 22465125 PMC3365616

[b28] Blaho V. A. & Hla T. Regulation of mammalian physiology, development, and disease by the sphingosine 1-phosphate and lysophosphatidic acid receptors. Chemical reviews 111, 6299–6320, doi: 10.1021/cr200273u (2011). 21939239 PMC3216694

[b29] Gomez-Munoz A. . Caged ceramide 1-phosphate (C1P) analogs: Novel tools for studying C1P biology. Chemistry and physics of lipids, doi: 10.1016/j.chemphyslip.2015.07.019 (2015). 26232662

[b30] Zheng S. . Sphingosine kinase 1 mediates neuroinflammation following cerebral ischemia. Exp Neurol 272, 160–169, doi: 10.1016/j.expneurol.2015.03.012 (2015). 25797575

[b31] Anderson D. M., Spraggins J. M., Rose K. L. & Schey K. L. High spatial resolution imaging mass spectrometry of human optic nerve lipids and proteins. Journal of the American Society for Mass Spectrometry 26, 940–947, doi: 10.1007/s13361-015-1143-9 (2015). 25893273 PMC5650057

[b32] Mechoulam R. & Parker L. A. The endocannabinoid system and the brain. Annual review of psychology 64, 21–47, doi: 10.1146/annurev-psych-113011-143739 (2013). 22804774

[b33] Di Marzo V., Stella N. & Zimmer A. Endocannabinoid signalling and the deteriorating brain. Nature reviews. Neuroscience 16, 30–42, doi: 10.1038/nrn3876 (2015). 25524120 PMC4471876

[b34] Nomura D. K. . Endocannabinoid hydrolysis generates brain prostaglandins that promote neuroinflammation. Science (New York, N.Y.) 334, 809–813, doi: 10.1126/science.1209200 (2011). PMC3249428 22021672

[b35] Panikashvili D. . An endogenous cannabinoid (2-AG) is neuroprotective after brain injury. Nature 413, 527–531, doi: 10.1038/35097089 (2001). 11586361

[b36] Serhan C. N., Dalli J., Colas R. A., Winkler J. W. & Chiang N. Protectins and maresins: New pro-resolving families of mediators in acute inflammation and resolution bioactive metabolome. Biochimica et biophysica acta 1851, 397–413, doi: 10.1016/j.bbalip.2014.08.006 (2015). 25139562 PMC4324013

[b37] Belayev L. . Docosahexaenoic Acid therapy of experimental ischemic stroke. Transl Stroke Res 2, 33–41, doi: 10.1007/s12975-010-0046-0 (2011). 21423332 PMC3037476

[b38] Gregersen R., Lambertsen K. & Finsen B. Microglia and macrophages are the major source of tumor necrosis factor in permanent middle cerebral artery occlusion in mice. Journal of cerebral blood flow and metabolism: official journal of the International Society of Cerebral Blood Flow and Metabolism 20, 53–65, doi: 10.1097/00004647-200001000-00009 (2000). 10616793

[b39] Huang W. C., Nagahashi M., Terracina K. P. & Takabe K. Emerging Role of Sphingosine-1-phosphate in Inflammation, Cancer, and Lymphangiogenesis. Biomolecules 3, doi: 10.3390/biom3030408 (2013). PMC3839861 24286034

[b40] Inose Y., Kato Y., Kitagawa K., Uchiyama S. & Shibata N. Activated microglia in ischemic stroke penumbra upregulate MCP-1 and CCR2 expression in response to lysophosphatidylcholine derived from adjacent neurons and astrocytes. Neuropathology: official journal of the Japanese Society of Neuropathology 35, 209–223, doi: 10.1111/neup.12182 (2015). 25443158

[b41] Hansen H. S., Moesgaard B., Hansen H. H. & Petersen G. N-Acylethanolamines and precursor phospholipids - relation to cell injury. Chemistry and physics of lipids 108, 135–150 (2000). 11106787 10.1016/s0009-3084(00)00192-4

[b42] Moesgaard B., Jaroszewski J. W. & Hansen H. S. Accumulation of N-acyl-ethanolamine phospholipids in rat brains during post-decapitative ischemia: a P-31 NMR study. Journal of lipid research 40, 515–521 (1999). 10064740

[b43] Hansen H. H., Hansen S. H., Schousboe A. & Hansen H. S. Determination of the phospholipid precursor of anandamide and other N-acylethanolamine phospholipids before and after sodium azide-induced toxicity in cultured neocortical neurons. Journal of neurochemistry 75, 861–871, doi: 10.1046/j.1471-4159.2000.0750861.x (2000). 10899965

[b44] Roux A. . Mass spectrometry imaging of rat brain lipid profile changes over time following traumatic brain injury. Journal of neuroscience methods, doi: 10.1016/j.jneumeth.2016.02.004 (2016). PMC7577232 26872743

[b45] Hannun Y. A. A personal journey with bioactive lipids. European Journal of Lipid Science and Technology 117, 1814–1831, doi: 10.1002/ejlt.201500135 (2015).

[b46] Saito M. . Involvement of ceramide in ethanol-induced apoptotic neurodegeneration in the neonatal mouse brain. Journal of neurochemistry 115, 168–177, doi: 10.1111/j.1471-4159.2010.06913.x (2010). 20663015 PMC2939968

[b47] Gomez-Munoz A. . Control of inflammatory responses by ceramide, sphingosine 1-phosphate and ceramide 1-phosphate. Progress in lipid research, doi: 10.1016/j.plipres.2015.09.002 (2015). 26703189

[b48] Galvani S. . HDL-bound sphingosine 1-phosphate acts as a biased agonist for the endothelial cell receptor S1P1 to limit vascular inflammation. Science signaling 8, ra79, doi: 10.1126/scisignal.aaa2581 (2015). 26268607 PMC4768813

[b49] Shohami E., Cohen-Yeshurun A., Magid L., Algali M. & Mechoulam R. Endocannabinoids and traumatic brain injury. British journal of pharmacology 163, 1402–1410, doi: 10.1111/j.1476-5381.2011.01343.x (2011). 21418185 PMC3165950

[b50] Zarruk J. G. . Cannabinoid Type 2 Receptor Activation Downregulates Stroke-Induced Classic and Alternative Brain Macrophage/Microglial Activation Concomitant to Neuroprotection. Stroke; a journal of cerebral circulation 43, 211–U413, doi: 10.1161/strokeaha.111.631044 (2012). 22020035

[b51] Higuchi S. . Reducing acyl migration during purification of 2-arachidonoylglycerol from biological samples before gas chromatography mass spectrometry analysis. Analytical sciences: the international journal of the Japan Society for Analytical Chemistry 26, 1199–1202 (2010). 21079352 10.2116/analsci.26.1199

[b52] Viader A. . Metabolic Interplay between Astrocytes and Neurons Regulates Endocannabinoid Action. Cell Reports 12, 798–808, doi: 10.1016/j.celrep.2015.06.075 (2015). 26212325 PMC4526356

[b53] Back M. . Update on leukotriene, lipoxin and oxoeicosanoid receptors: IUPHAR Review 7. British journal of pharmacology 171, 3551–3574, doi: 10.1111/bph.12665 (2014). 24588652 PMC4128057

[b54] Murphy R. C., Hankin J. A. & Barkley R. M. Imaging of lipid species by MALDI mass spectrometry. Journal of lipid research 50 Suppl, S317–322, doi: 10.1194/jlr.R800051-JLR200 (2009). 19050313 PMC2674737

[b55] Hattori K. . Paradoxical ATP Elevation in Ischemic Penumbra Revealed by Quantitative Imaging Mass Spectrometry. Antioxid. Redox Signal. 13, 1157–1167, doi: 10.1089/ars.2010.3290 (2010). 20486758 PMC2956403

[b56] Morikawa T. . Hypoxic regulation of the cerebral microcirculation is mediated by a carbon monoxide-sensitive hydrogen sulfide pathway. Proceedings of the National Academy of Sciences of the United States of America 109, 1293–1298, doi: 10.1073/pnas.1119658109 (2012). 22232681 PMC3268316

[b57] Sugiura Y., Honda K., Kajimura M. & Suematsu M. Visualization and quantification of cerebral metabolic fluxes of glucose in awake mice. Proteomics 14, 829–838, doi: 10.1002/pmic.201300047 (2014). 23970501

[b58] Saghatelian A., McKinney M. K., Bandell M., Patapoutian A. & Cravatt B. F. A FAAH-regulated class of N-acyl taurines that activates TRP ion channels. Biochemistry 45, 9007–9015, doi: 10.1021/bi0608008 (2006). 16866345

[b59] Chatzakos V., Slatis K., Djureinovic T., Helleday T. & Hunt M. C. N-Acyl Taurines are Anti-Proliferative in Prostate Cancer Cells. Lipids 47, 355–361, doi: 10.1007/s11745-011-3639-9 (2012). 22160494

[b60] Thunig J., Hansen S. H. & Janfelt C. Analysis of secondary plant metabolites by indirect desorption electrospray ionization imaging mass spectrometry. Analytical chemistry 83, 3256–3259, doi: 10.1021/ac2004967 (2011). 21473636

[b61] Bouschen W., Schulz O., Eikel D. & Spengler B. Matrix vapor deposition/recrystallization and dedicated spray preparation for high-resolution scanning microprobe matrix-assisted laser desorption/ionization imaging mass spectrometry (SMALDI-MS) of tissue and single cells. Rapid communications in mass spectrometry: RCM 24, 355–364, doi: 10.1002/rcm.4401 (2010). 20049881

[b62] Schramm T. . imzML — A common data format for the flexible exchange and processing of mass spectrometry imaging data. Journal of Proteomics 75, 5106–5110, doi: 10.1016/j.jprot.2012.07.026 (2012). 22842151

[b63] Robichaud G., Garrard K. P., Barry J. A. & Muddiman D. C. MSiReader: An Open-Source Interface to View and Analyze High Resolving Power MS Imaging Files on Matlab Platform. Journal of the American Society for Mass Spectrometry 24, 718–721, doi: 10.1007/s13361-013-0607-z (2013). 23536269 PMC3693088

