# Supplementary material for: Mass spectrometry imaging of biomarker lipids for phagocytosis and signalling during focal cerebral ischaemia
Source: Sci Rep. 2016 Dec 22;6:39571. doi: 10.1038/srep39571 (PMC5177920; doi:10.1038/srep39571)
Supplement: Supplementary Information [file srep39571-s1.pdf]

Supplementary Information

Mass spectrometry imaging of biomarker lipids for phagocytosis and signalling during focal cerebral ischaemia

Mette M. B. Nielsen[1], Kate L. Lambertsen[2], Bettina H. Clausen[2], Morten Meyer[2], Dhaka R. Bhandari[3], Søren T. Larsen[4], Steen S. Poulsen[5], Bernhard Spengler[3], Christian Janfelt[6] & Harald S. Hansen[1*]

[1]Department of Drug Design and Pharmacology, University of Copenhagen, Universitetsparken 2, DK-2100 Copenhagen, Denmark.

[2]Department of Neurobiology Research, University of Southern Denmark, J. B. Winsløws Vej 21, DK-5000, Odense, Denmark.

[3]Institute of Inorganic and Analytical Chemistry, Justus Liebig University, Heinrich-Buff-Ring 17, D-35392, Giessen, Germany.

[4]National Research Centre for the Working Environment, Lersø Parkallé 105, DK-2100, Copenhagen, Denmark. [5]Department of Biomedical Sciences, University of Copenhagen, Blegdamsvej 3, DK-2200, Copenhagen, Denmark. [6]Department of Pharmacy, University of Copenhagen, Universitetsparken 2, DK-2100, Copenhagen, Denmark. Correspondence and requests for materials should be addressed to H.S.H. (email: hsh@sund.ku.dk).

Supplementary Figures

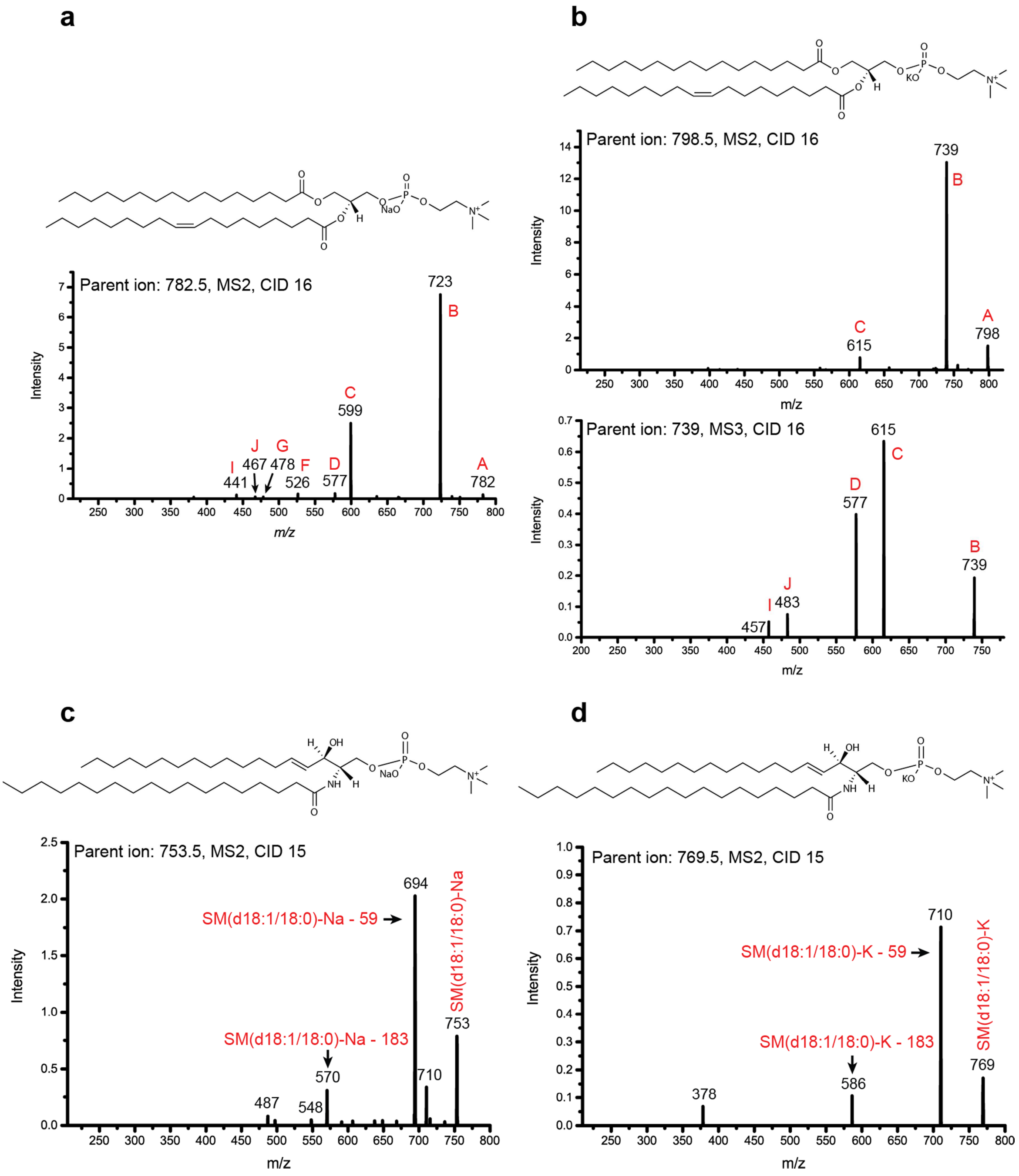

Supplementary Figure S1. Molecular information about PC(16:0/18:1) and SM(d18:1/18:0): **(a)** Molecular structure of the PC(16:0/18:1) sodium adduct ion and a typical MS/MS mass spectrum for *m/z* 782.5. **(b)** Molecular structure of the PC(16:0/18:1) potassium adduct ion and its MS/MS and MS^3 mass spectra at *m/z* 798.5 and *m/z* 739 respectively. **(c)** Molecular structure of the SM(d18:1/18:0) sodium adduct ion and its MS/MS mass spectrum at *m/z* 753.5. **(d)** Molecular structure of the SM(d18:1/18:0) potassium

adduct ion and its MS/MS spectrum at *m/z* 769.5. The corresponding molecular structures to the assigned letters in the spectra can be seen in Supplementary Table S1.

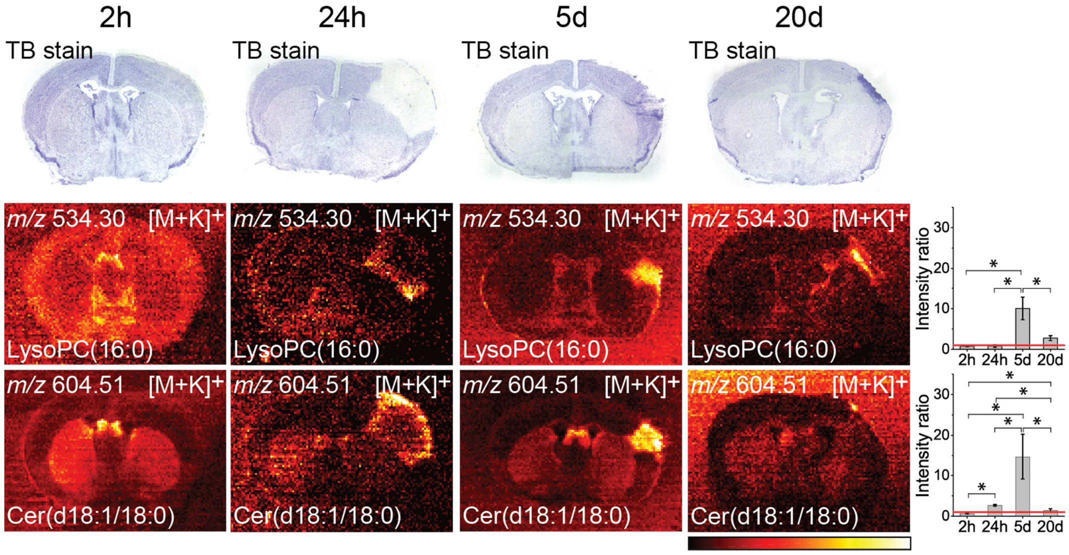

Supplementary Figure S2. Potassium adducts of LysoPC(16:0) and Cer(d18:1/18:0): The accumulation of the potassium adducts of lysoPC(16:0) and Cer(d18:1/18:0) over time show the same behaviour as the sodium adducts of lysoPC(16:0) and Cer(d18:1/18:0) respectively, which can be seen in Fig. 1. The ion images have individual intensity bars between 0-100%, and therefore, the intensity colours cannot be compared between two images. For each lipid, the ratio between the intensity of the ischaemic area and the comparable size area in the contralateral site are shown on the right side where bars are mean±SEM (n=3), * $p< 0.05$. The red line indicates ratio = one. All images were measured in positive ion mode by DESI imaging with a spatial resolution of 100x100 μm^2 and the images are a typical representative of 3 mice.

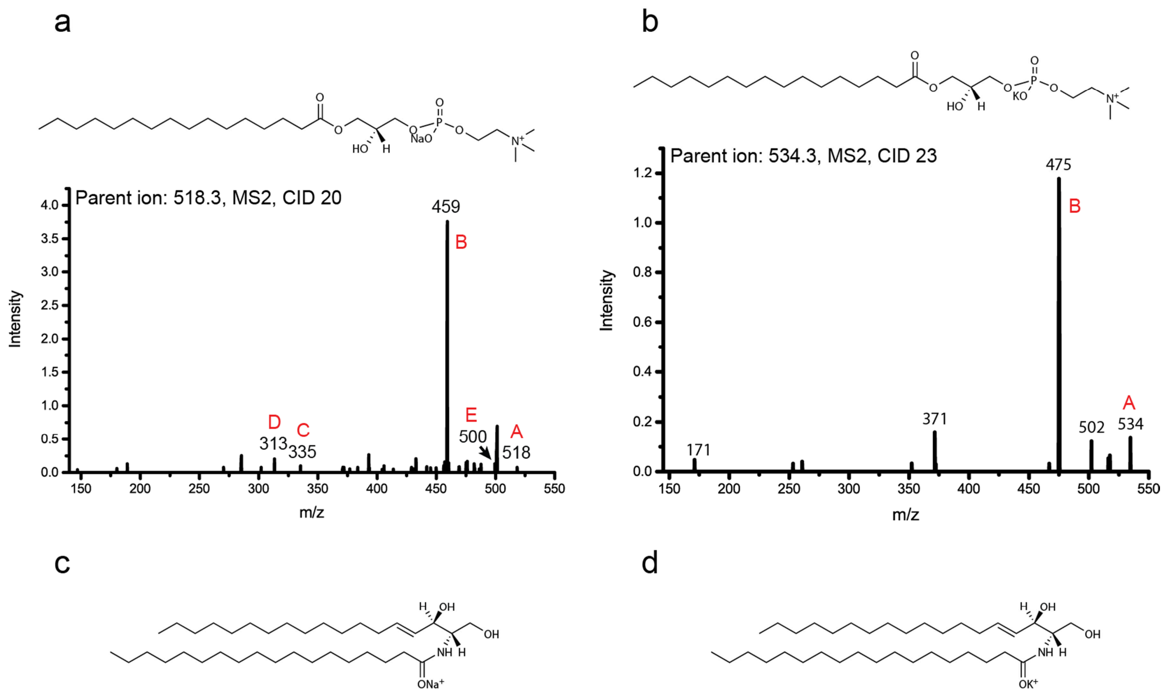

Supplementary Figure S3. Molecular information about LysoPC(16:0) and Cer(d18:1/18:0): **(a)** Molecular structure of the LysoPC(16:0) sodium adduct ion and its MS/MS spectrum at *m/z* 518.3. **(b)** Molecular structure of the LysoPC(16:0) potassium adduct ion and its MS/MS spectrum at *m/z* 534.3. **(c)** Molecular structure of the Cer(d18:1/18:0) sodium adduct and **(d)** molecular structure of the Cer(d18:1/18:0) adduct. For **(c)** and **(d)** it was not possible to identify the molecular structure by MS/MS, however, we still believe that this in fact is Cer(d18:1/18:0) in accordance with results of others[1]. The corresponding molecular structures to the assigned letters in the spectra can be seen in Supplementary Table S1.

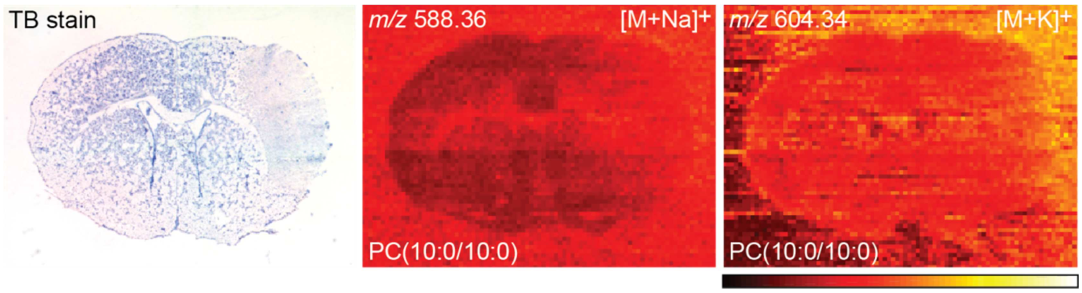

Supplementary Figure S4. PC(10:0/10:0) sprayed on section as internal standard: To test whether the ion suppression in the ischaemic area differed from the healthy brain tissue, we sprayed a section with 24h post-surgical survival with PC(10:0/10:0) (not naturally present in the brain). Especially the sodium adduct

but also to a lesser extent the potassium adduct for PC(10:0/10:0) seemed to accumulate more easily in the ischaemic area. However, this apparent smaller ion suppression in the ischaemic area appear not sufficient to explain the accumulation of e.g. LysoPC(16:0) and Cer(d18:1/18:0) during ischaemia. The ion images have individual intensity bars between 0-100%, and therefore, the intensity colours cannot be compared between two images. The images were measured in positive ion mode by DESI imaging with a spatial resolution of 120x120 μm^2 and the images are a typical representative of 2 mice.

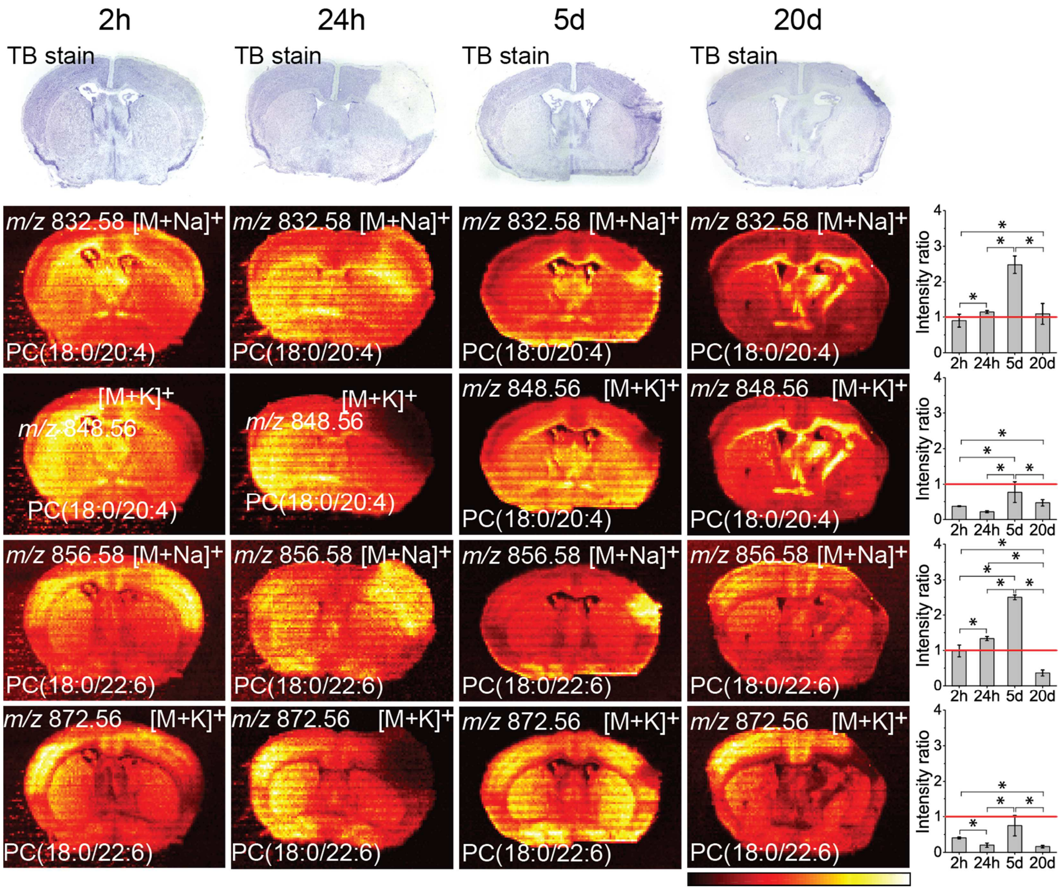

Supplementary Figure S5. AA- and DHA-rich PCs: The distribution of the sodium and potassium adducts of PC(18:0/20:4), which contains arachidonic acid (AA, 20:4(n-6)), and PC(18:0/22:6), which contains docosahexaenoic acid (DHA, 22:6(n-3)). The ion images have individual intensity bars between 0-100%, and therefore, the intensity colours cannot be compared between two images. For each lipid, the ratio between the intensity of the ischaemic area and the comparable size area in the contralateral site are shown on the right side where bars are mean±SEM (n=3), * $p < 0.05$. The red line indicates ratio = one. As in Fig. 1 both

PCs were affected by the breakdown of the Na^+/K^+-ATPase where the sodium adducts accumulated (although the accumulation were weaker for PC(18:0/20:4)), and the potassium adducts disappeared (see MS/MS spectra and molecular structures for PC(18:0/20:4) and PC(18:0/22:6) in Supplementary Fig. S6). All images were measured in positive ion mode by DESI imaging with a spatial resolution of 100x100 μm^2 and the images are a typical representative of 3 mice.

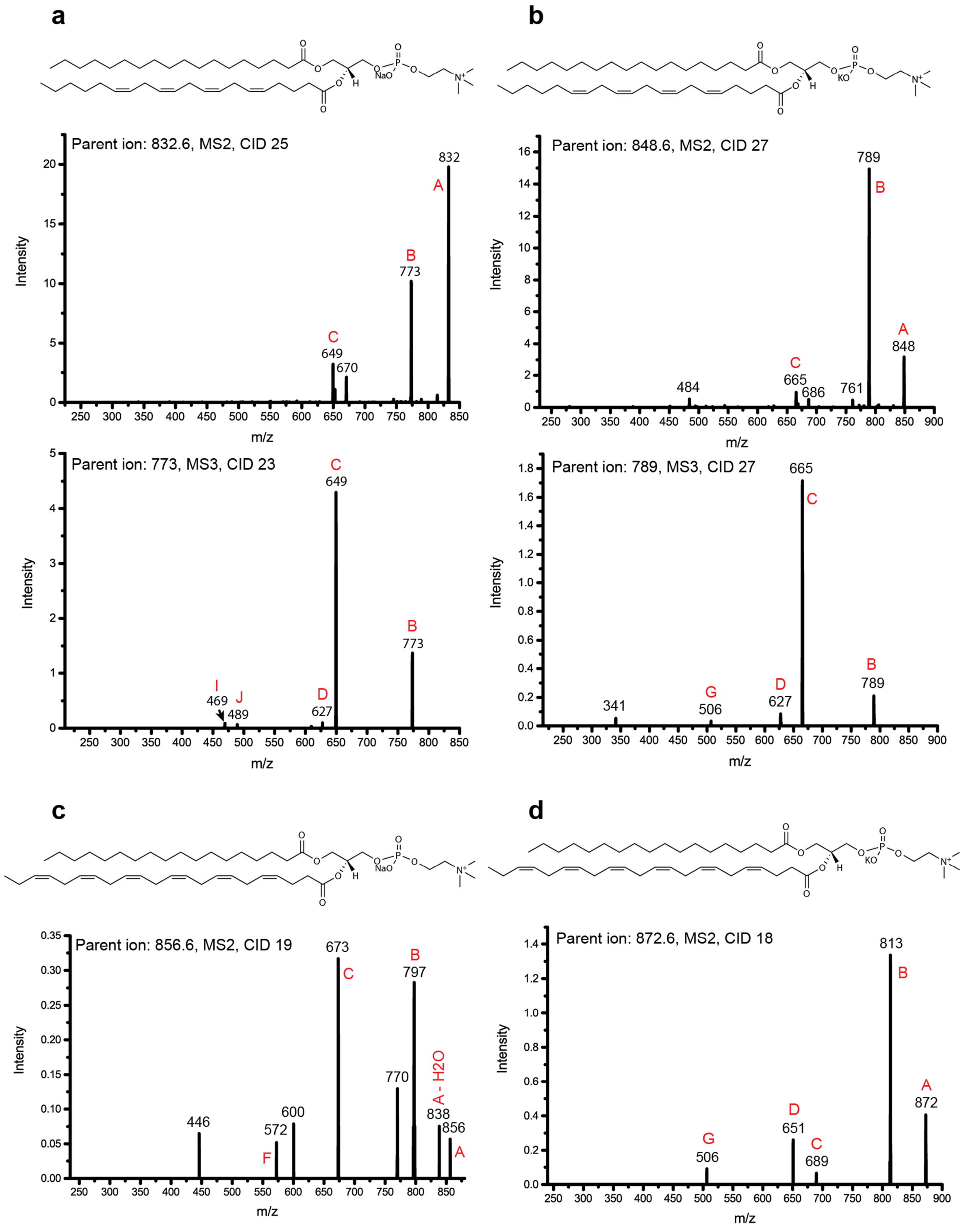

Supplementary Figure S6. Molecular information about PC(18:0/20:4) and PC(18:0/22:6): **(a)** Molecular structure of the PC(18:0/20:4) sodium adduct ion and its MS/MS and MS^3 its mass spectra at *m/z* 832.6 and *m/z* 773 respectively. **(b)** Molecular structure of the PC(18:0/20:4) potassium adduct and its

MS/MS and MS^3 mass spectra at *m/z* 848.6 and *m/z* 789 respectively. **(c)** Molecular structure of the PC(18:0/22:6) sodium adduct ion and its MS/MS mass spectrum at *m/z* 856.6. **(d)** Molecular structure of the PC(18:0/22:6) potassium adduct ion and its MS/MS mass spectrum at *m/z* 872.6. The corresponding molecular structures to the assigned letters in the spectra can be seen in Supplementary Table S1.

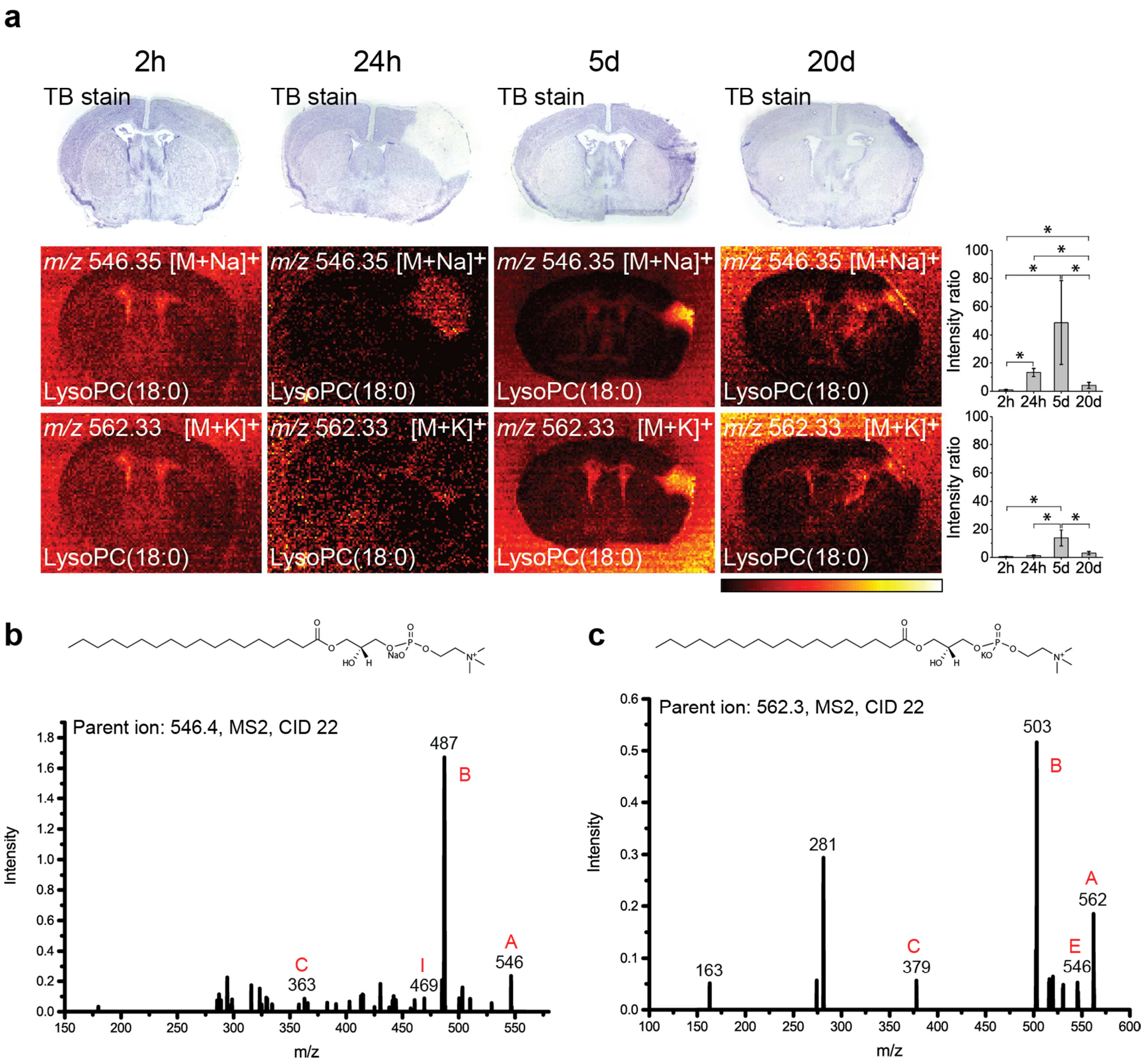

Supplementary Figure S7. Accumulation of LysoPC(18:0) and its molecular information: **(a)** The accumulation of the sodium and potassium adducts of LysoPC(18:0) over time show the same behaviour as the sodium and potassium adducts of LysoPC(16:0), which can be seen in Fig. 1 and Supplementary Fig. 2, respectively. The ion images have individual intensity bars between 0-100%, and therefore, the intensity colours cannot be compared between two images. For each lipid, the ratio between the intensity of the

ischaemic area and the comparable size area in the contralateral site are shown on the right side where bars are mean±SEM (n=3), * $p < 0.05$. All images were measured in positive ion mode by DESI imaging with a spatial resolution of 100x100 µm^2 and the images are a typical representative of 3 mice. **(b)** Molecular structure and MS/MS spectrum of the LysoPC(18:0) sodium adduct ion, and **(c)** molecular structure and MS/MS spectrum of the LysoPC(18:0) potassium adduct ion. The corresponding molecular structures to the assigned letters in the spectra can be seen in Supplementary Table S1.

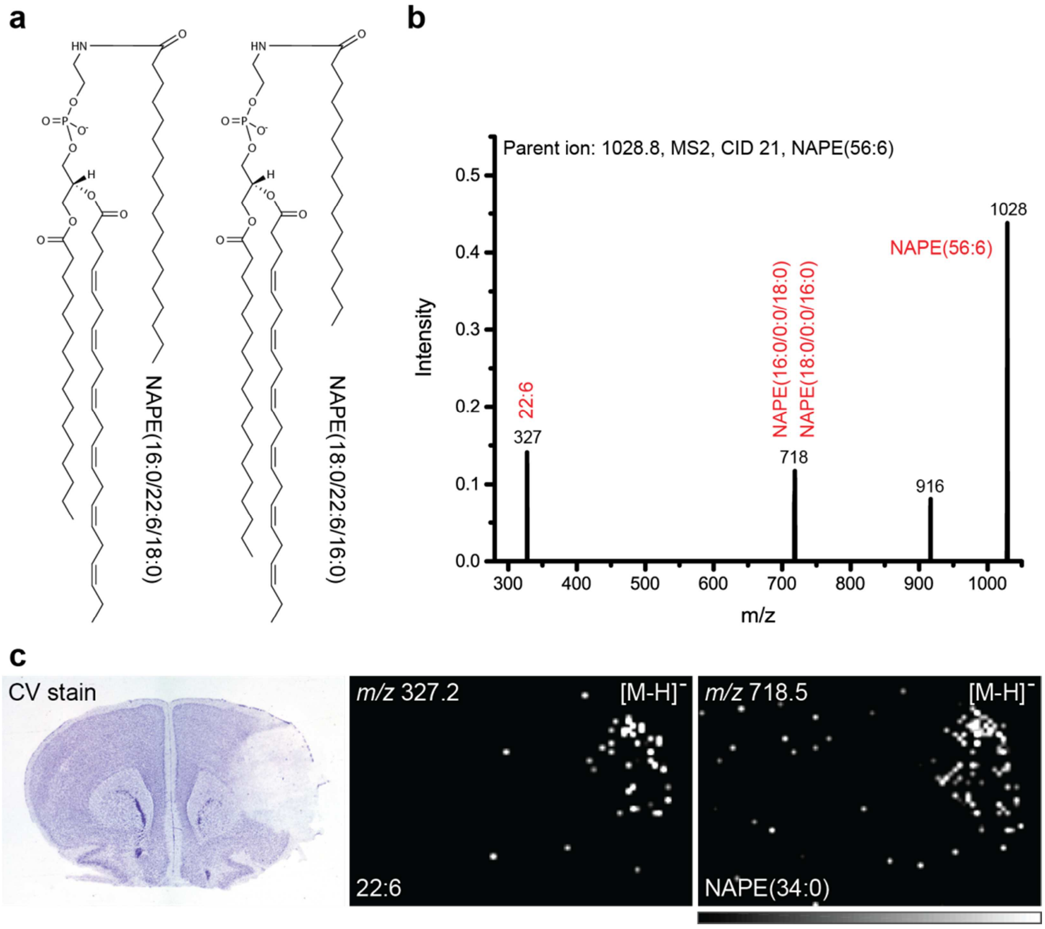

Supplementary Figure S8. Molecular information about NAPE(56:6). **(a)** The molecular structure of the NAPE(56:6) deprotonated ion could be either NAPE(16:0/22:6/18:0) or NAPE(18:0/22:6/16:0), as shown in **(b)** the MS/MS mass spectra. The accumulation of NAPE(56:6) is most likely consisting of both isomers. **(c)** The accumulation of two of the fragments (*m/z* 327.2 and *m/z* 718.5) by MS/MS is shown in a mouse brain section with 24h post-surgical survival. The images were measured in negative ion mode by DESI imaging with a spatial resolution of 120x120 µm^2.

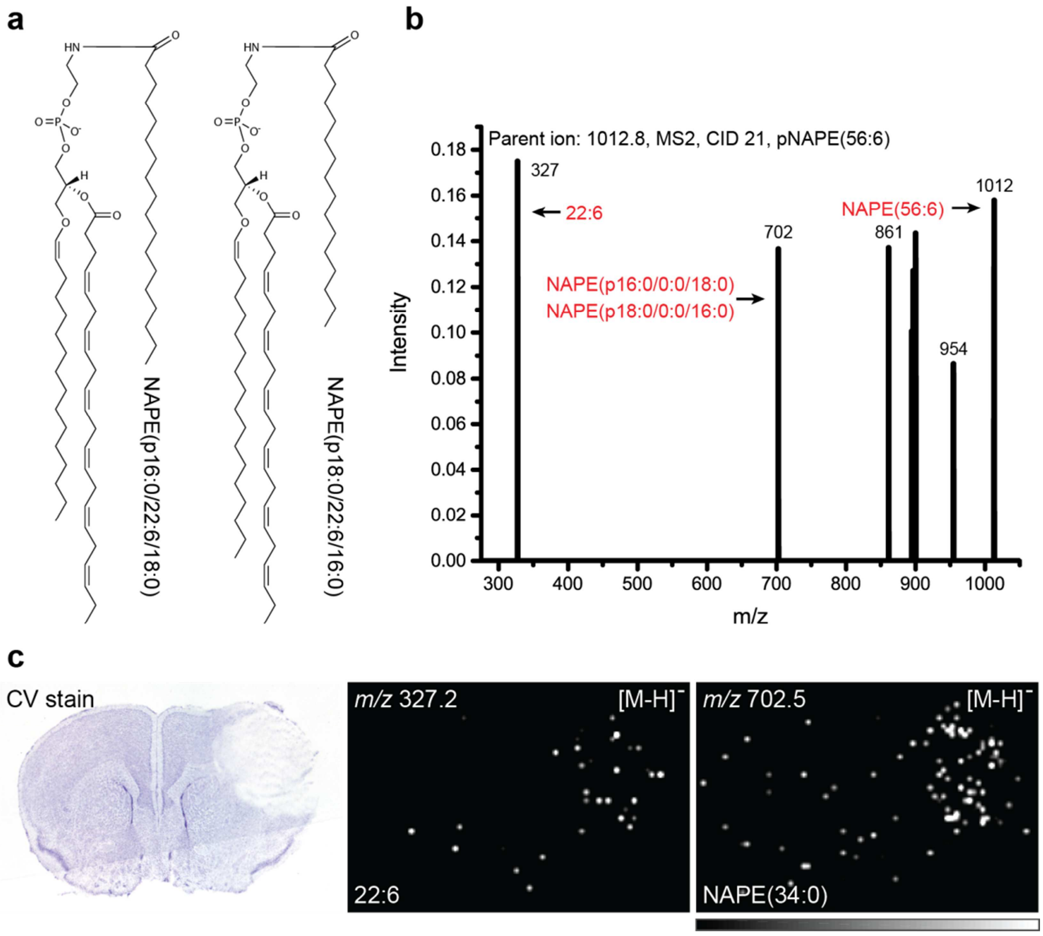

Supplementary Figure S9. Molecular information about pNAPE(56:6): **(a)** The molecular structure of the pNAPE(56:6) deprotonated ion could be either NAPE(p16:0/22:6/18:0) or NAPE(p18:0/22:6/16:0), as shown in **(b)** the MS/MS mass spectra. The accumulation of pNAPE(56:6) is most likely consisting of both isomers. **(c)** The accumulation of two of the fragments (*m/z* 327.2 and *m/z* 702.5) by MS/MS is shown in a mouse brain section with 24h post-surgical survival. The images were measured in negative ion mode by DESI imaging with a spatial resolution of 120x120 μm^2.

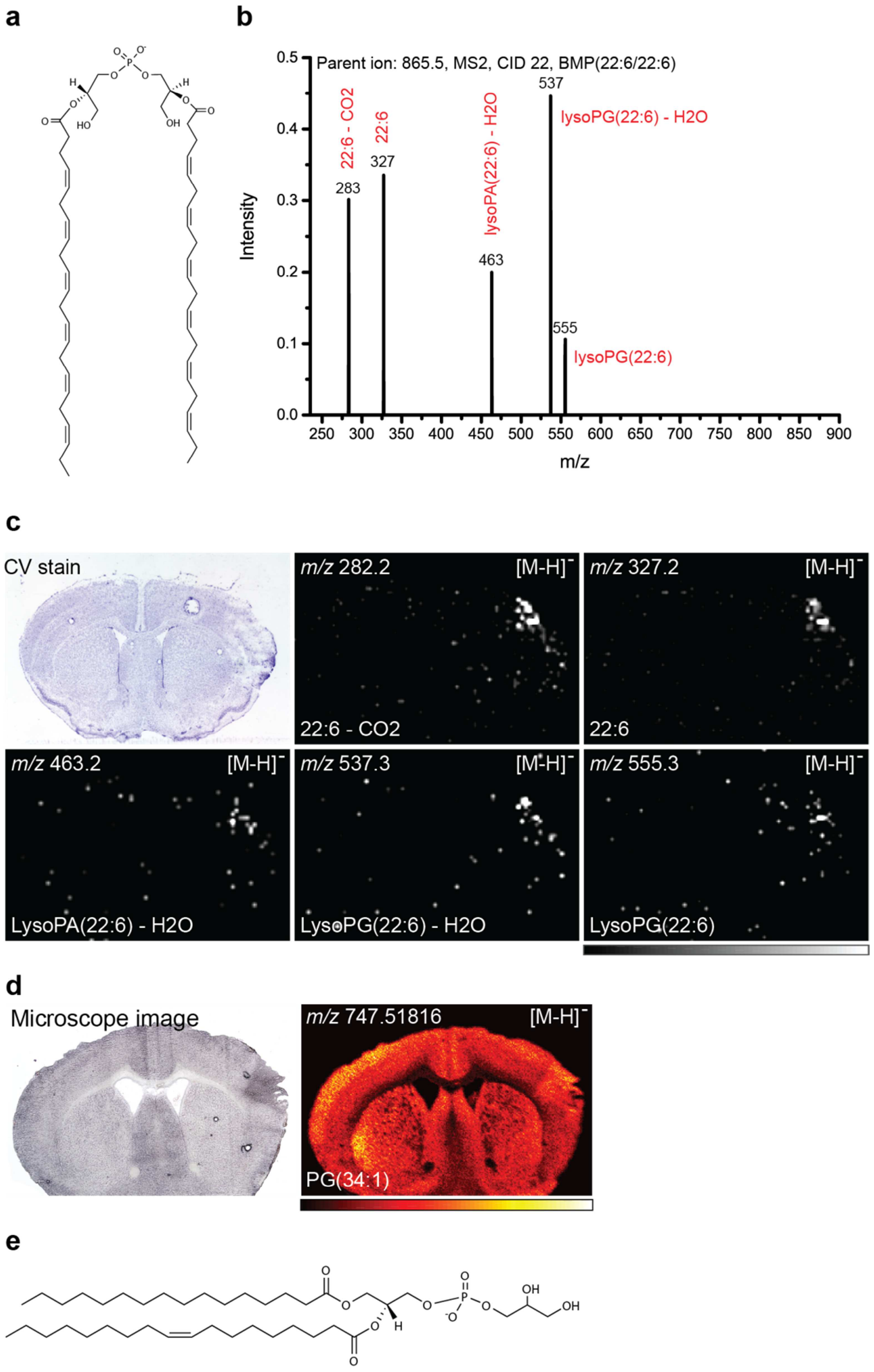

Supplementary Figure S10. Molecular information about BMP(22:6/22:6): **(a)** The molecular structure of the BMP(22:6/22:6) deprotonated ion and **(b)** its MS/MS mass spectra. **(c)** The accumulation of the

fragments by MS/MS is shown in a mouse brain section with 5d post-surgical survival. The images were measured in negative ion mode by DESI imaging with a spatial resolution of 120x120 μm^2. **(d)** The spatial distribution of PG(34:1) at 5d post-surgical survival measured in negative ion mode by MALDI imaging with a spatial resolution of 35x35 μm^2 and the image is a typical representative of 3 mice. **(e)** The molecular structure of the PG(34:1) deprotonated ion (here shown as PG(16:0/18:1). The fragments of BMP(22:6/22:6) and PG(22:6/22:6) are identical as the two lipids are isomers. However, we believe that the accumulation at *m/z* 865.5 comes from BMP(22:6/22:6) since it is the most abundant BMP species in the mouse brain (50 to 70%), while the most abundant PG species, PG(34:1), account for over 90% of PG species in the mouse brain[2]. The total BMP and the total PG in the brain is of the same magnitude[2].

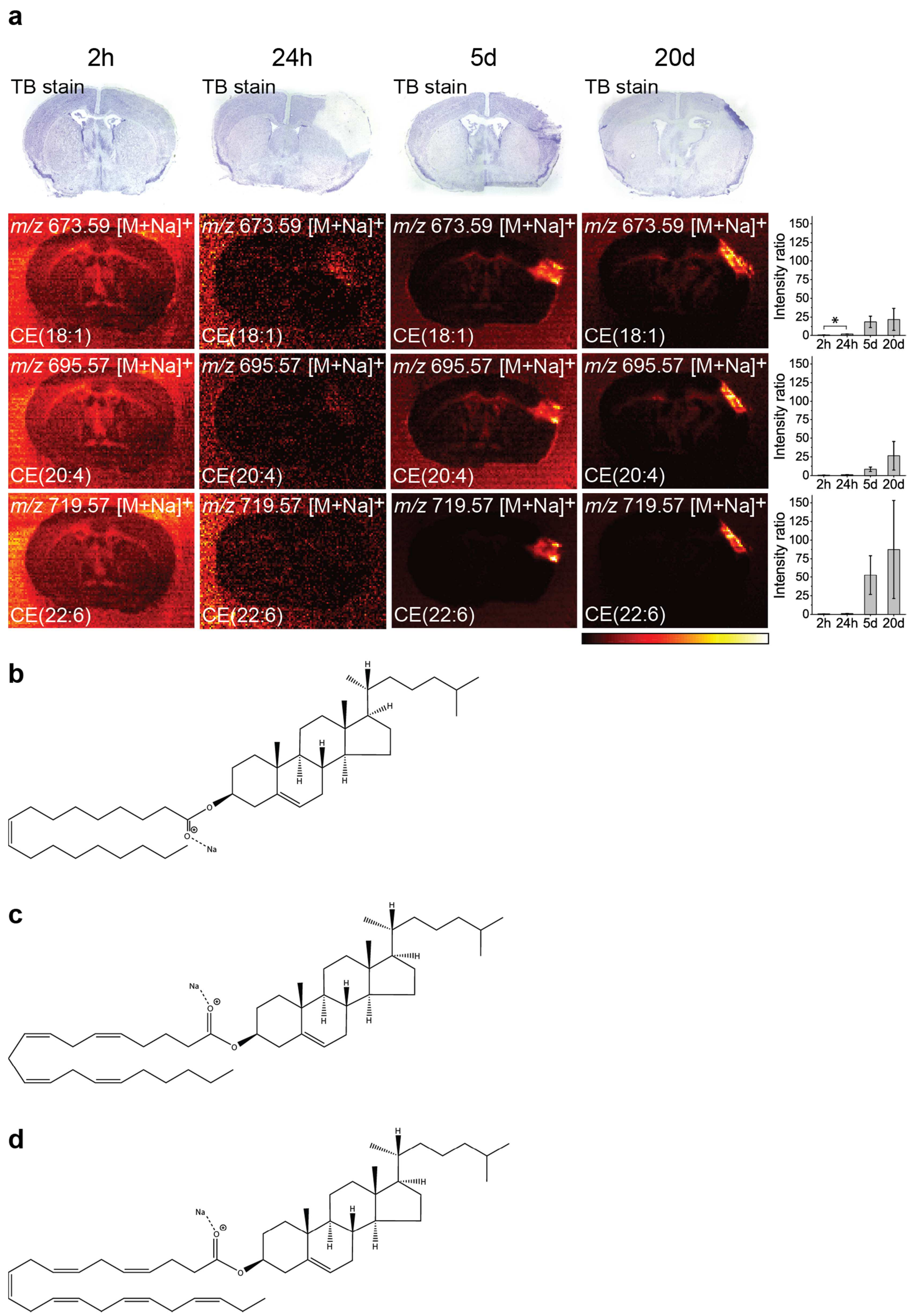

Supplementary Figure S11. Accumulation of CEs and their molecular structure: **(a)** Accumulation of CE(18:1), CE(20:4), and CE(22:6) in the ischaemic area is observed weakly at 24h, strongly at 5d and 20d. The ion images have individual intensity bars between 0-100%, and therefore, the intensity colours cannot be

compared between two images. For each lipid, the ratio between the intensity of the ischaemic area and the comparable size area in the contralateral site are shown on the right side where bars are mean±SEM (n=3), * $p< 0.05$. The images were measured in positive ion mode by DESI imaging with a spatial resolution of 100x100 μm^2 and the images are a typical representative of 3 mice. Molecular structure of the **(b)** CE(18:1) sodium adduct ion, **(c)** CE(20:4) sodium adduct ion, and **(d)** CE(22:6) sodium adduct ion.

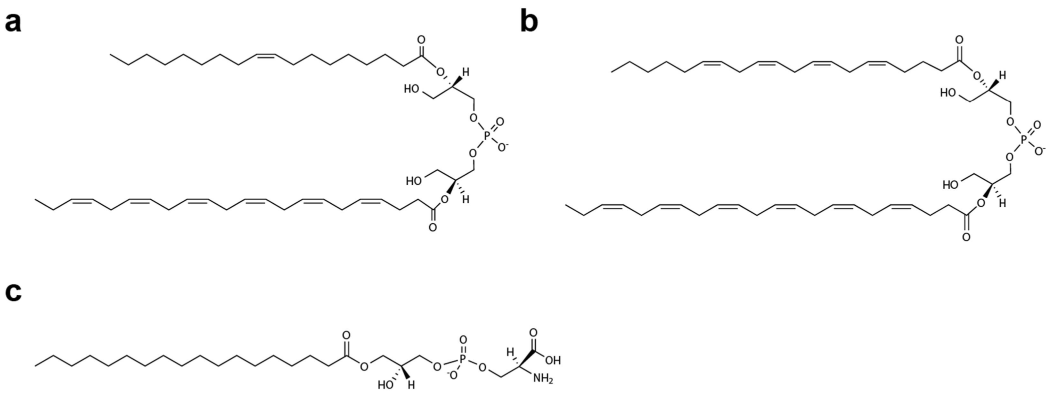

Supplementary Figure S12. Molecular structure of BMP(40:7), BMP(42:10), and LysoPS(18:0): Molecular structure of the **(a)** BMP(40:7) deprotonated ion (here shown as BMP(18:1/22:6)), **(b)** BMP(42:10) deprotonated ion (here shown as BMP(20:4/22:6)), and **(c)** LysoPS(18:0) deprotonated ion.

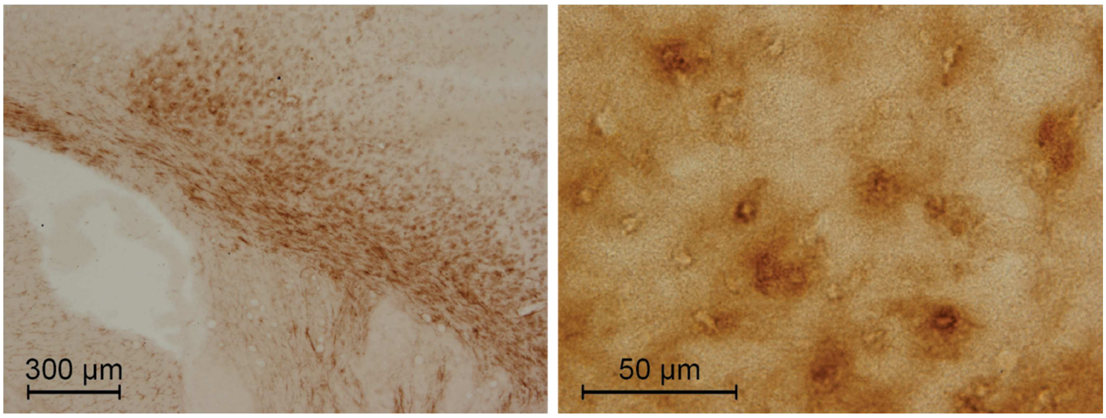

Supplementary Figure S13. Immunohistochemistry for macrophage/microglia using CD11b labelling: High magnification photomicrographs of a brain section from a mouse subjected to pMCAO with 5 days post-surgical survival and stained for CD11b.

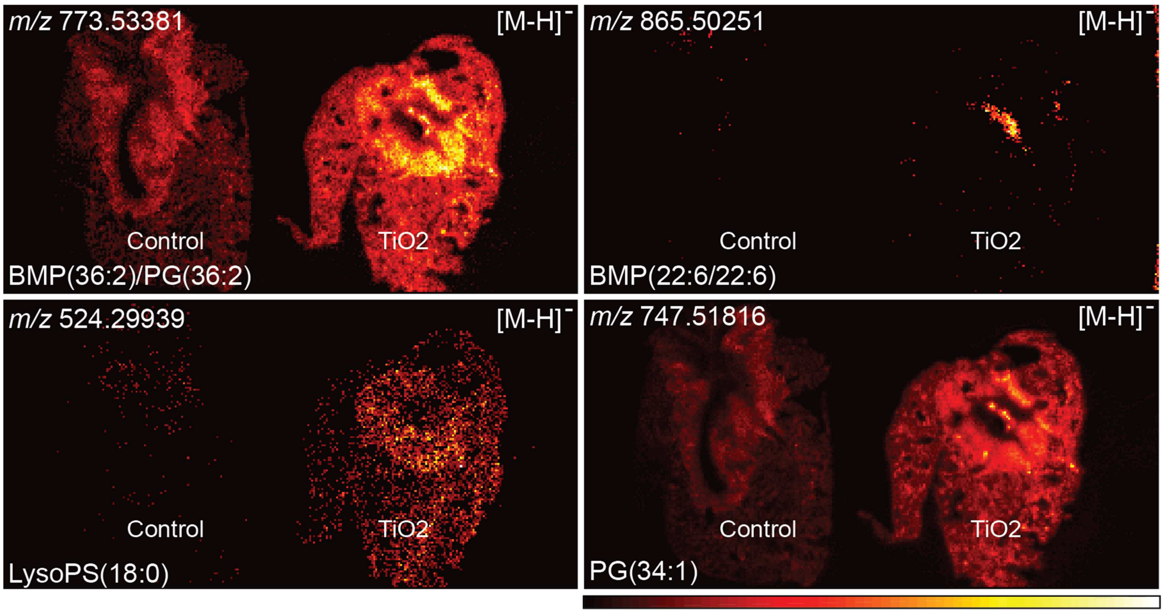

Supplementary Figure S14. Accumulation of BMP and LysoPS in TiO_2-nanoparticle-exoposed mouse lungs: BMP is especially abundant in alveolar macrophages and we, therefore, analysed the distribution of BMP species in control mouse lungs and TiO_2-nanoparticle-exposed mouse lungs. This showed that BMP(36:2) and BMP(22:6/22:6) were increased in the TiO_2- nanoparticle-exposed lungs compared to the controls. Furthermore, LysoPS(18:0) had also increased in the TiO_2- nanoparticle-exposed lungs. PG(34:1) also seemed to be increased in the TiO_2- nanoparticle-exposed lungs compared to the control, however, not to the same extent. The ion images have individual intensity bars between 0-100%, and therefore, the intensity colours cannot be compared between two images. The images were measured in negative ion mode by MALDI imaging with a spatial resolution of 50x50 μm^2 and are a typical representative of 3 mice. BMP and PG are isomeric molecules that can be difficult to differentiate by MS/MS[3]. However, mouse lung surfactant obtained from bronchoalveolar lavage consist mainly of PC and a minor percentage of PG, of which the PG(34:1) is the major species and PG(36:2) accounts to only a few percentage of the PG species[4] and PG(44:12) was not found[4]. Therefore, we have assigned the *m/z*-value 865.50251 as being BMP(22:6/22:6) and the *m/z*-value 747.51816 as being PG(34:1). The *m/z*-value of 773.53381 may be a mixture of BMP(36:2) and PG(36:2) where the higher density areas of *m/z* 773.53381 co-localizing and surrounding the area with *m/z*-value 865.50251 may mainly represent BMP(36:2).

Supplementary Tables

Fragment	Sodium adduct	Potassium adduct
A	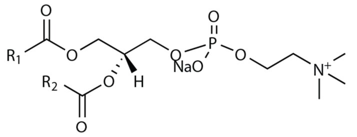	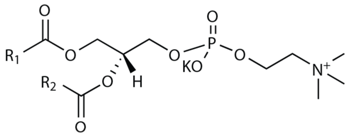
B	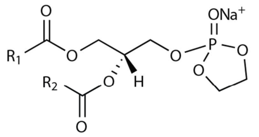	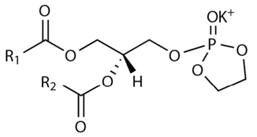
C	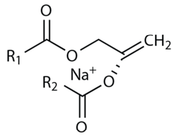	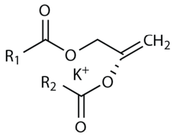
D	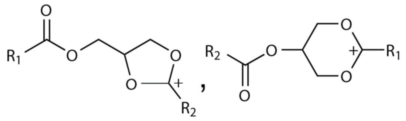	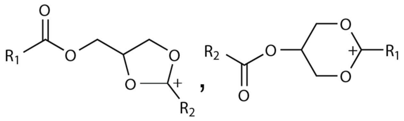
E	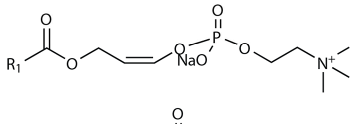	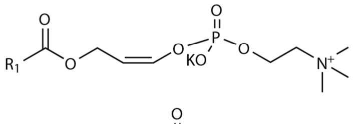
F	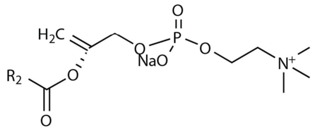	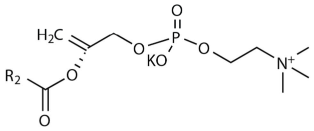
G	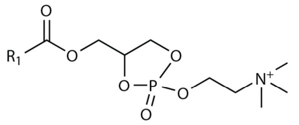	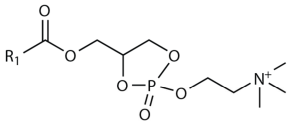
I	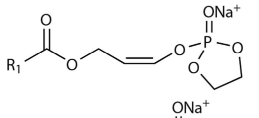	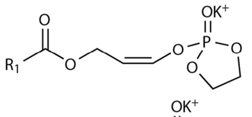
J	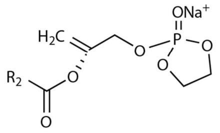	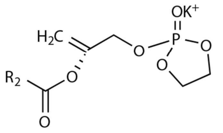

Supplementary Table S1. PC fragments for the sodium and potassium adduct: MS/MS and MS^3 molecular structures of fragments for PC species. The letters are assigned to its corresponding peaks in the MS/MS and MS^3 spectra for the different PCs investigated in this article. The structures are based on the suggestions by Al-Saad K. A et al[5].

Supplementary Methods

MS/MS and MS^3 measurements on mouse brains. MS/MS and MS^3 were carried out on mouse brains to identify the molecular structures of lipid species using DESI on a LTQ XL linear ion trap mass spectrometer (Thermo Scientific, USA) equipped with a custom-built DESI imaging ion source, as previously described[6]. An *m/z* value of interest was separated and fragmented by Collision-Induced Dissociation (CID) (MS/MS). Using the *m/z* values of the fragments, the molecular structure could be identified. In some cases the most abundant fragment was separated and then fragmented by CID giving further molecular structure information (MS^3). For some lipids the spatial localization of their CID fragments were investigated by DESI imaging, which was carried out as described in the method section.

Spraying section with lipid standard. A Brain section with 24h post-surgical survival was placed on a moving stage and with a spray pointed directly towards the section with a spray-to-sample distance of 3 cm. The spray was constructed of coaxial fused silica capillaries connected in 1/16-inch Swagelok tee (Swagelok Co., USA), an inner capillary (50 µm ID, 150 µm OD, SGE, USA) carrying the spray solvent, and an outer capillary (250 µm ID, 350 µm OD, SGE, USA) carrying the nebulizer gas. The spray consisted of 0.01 mg/mL PC(10:0/10:0) in methanol and water (95:5) dispensed with a flow of 10 µl/min and a nitrogen nebulizer gas pressure of 2 bar. The section was moved under the spray with a speed of 10 mm/s in a meandering pattern across the section and this was repeated over 10 cycles. Afterwards the Section was measured by DESI imaging as described in the method section.

Cresyl violet acetate stains. Sections were after end of the measurement stained with 0.5% Cresyl violet acetate (Sigma-Aldrich, USA) in water for approximately 8 min. and then dehydrated in graded series of alcohol (70-99%), cleared in xylene, and finally cover-slipped with Eukitt quick-hardening mounting medium (Fluka Analytical, Sigma-Aldrich, Missouri, USA).The Cresyl violet acetate (CV) stains were then captured on a Stemi DV4 Stereoscope (Carl Zeiss AG, Oberkuchen, Germany) equipped with an LCMOS digital streaming camera (Brunel Microscopes Ltd, Chippenham, UK). Images composed of more than one image were stitched by the Image Composite Editor (Microsoft Corporation, Washington, USA).

Animals for Lung measurements. Inbred female BALB/cJ mice aged 6-7 weeks were purchased from Taconic M&B, Ry, Denmark, and were housed in polypropylene cages (380×220×150mm) with pinewood sawdust bedding (Lignocel S8, Brogaarden, Denmark). The cages were furnished with bedding materials, gnaw sticks and cardboard tubes. The photo-period was from 6 a.m. to 6 p.m., and the temperature and mean relative humidity in the animal room were 19-22°C and 43 ± 8% (SD), respectively. Food (Altromin no. 1324, Altromin, Lage, Germany) and tap water were available *ad libitum*. All experiments followed relevant procedures and guidelines by the Animal Experiment Inspectorate, Denmark. Experiments were approved by the Animal Experiment Inspectorate, Denmark (permission 2014-15-2934-01042). At the time of exposure, the mice had a body weight of 21.6±0.8 g (mean±SD).

Generation of TiO_2 aerosol. Mice were exposed head-only to an aerosol of TiO_2 (PlasmaChem GmbH, Berlin, Germany) at a concentration of 330 mg/m^3 for 30 min. The particle had a primary size of 15 nm as measured by aerodynamic particle sizer and a specific surface area of 98 m^2/g (Brunauer-Emmett-Teller (BET) nitrogen adsorption method). The particles were aerosolized using a dry powder aerosol generator (Microdosing system, Fraunhofer ITEM, Hannover, Germany). The generator was operated at a pressure of 1.0 bar which generated an airflow of 14.7 L/min. Aerosols were continuously collected from the breathing zone of the mice throughout all exposure studied and total aerosol mass concentrations were determined gravimetrically by a filter sampling protocol[7].

Tissue preparation. The TiO_2-nanoparticle-exposed mice were after 24h euthanized along with a control group of non-exposed mice (3 mice in each group) followed by removal of the lungs. The left lung of each mouse were then frozen and subsequently cut into 30 µm thick cryostat sections. Sections were placed on microscope slides and stored in sealed boxes at -80° C. Before MALDI imaging (described in method section) a section was removed from the freezer and placed in a vacuum desiccator for approximately 10 min. to remove water and thus prevent enzymatic reactions in the lung tissue during the measurement.

Supplementary References

1 Wang, H. Y., Wu, H. W., Tsai, P. J. & Liu, C. B. MALDI-mass spectrometry imaging of desalted rat brain sections reveals ischemia-mediated changes of lipids. *Analytical and bioanalytical chemistry* **404**, 113-124, doi:10.1007/s00216-012-6077-5 (2012).

2 Saville, J. T., Lehmann, R. J., Derrick-Roberts, A. L. K. & Fuller, M. Selective normalisation of regional brain bis(monoacylglycero)phosphate in the mucopolysaccharidosis 1 (Hurler) mouse. *Exp. Neurol.* **277**, 68-75, doi:10.1016/j.expneurol.2015.12.012 (2016).

3 Hankin, J. A., Murphy, R. C., Barkley, R. M. & Gijon, M. A. Ion mobility and tandem mass spectrometry of phosphatidylglycerol and bis(monoacylglycerol) phosphate (BMP). *International Journal of Mass Spectrometry* **378**, 255-263, doi:10.1016/j.ijms.2014.08.026 (2015).

4 Herber-Jonat, S. *et al.* Abca3 haploinsufficiency is a risk factor for lung injury induced by hyperoxia or mechanical ventilation in a murine model. *Pediatric Research* **74**, 384-392, doi:10.1038/pr.2013.127 (2013).

5 Al-Saad, K. A., Siems, W. F., Hill, H. H., Zabrouskov, V. & Knowles, N. R. Structural analysis of phosphatidylcholines by post-source decay matrix-assisted laser desorption/ionization time-of-flight mass spectrometry. *Journal of the American Society for Mass Spectrometry* **14**, 373-382, doi:10.1016/s1044-0305(03)00068-0 (2003).

6 Thunig, J., Hansen, S. H. & Janfelt, C. Analysis of secondary plant metabolites by indirect desorption electrospray ionization imaging mass spectrometry. *Analytical chemistry* **83**, 3256-3259, doi:10.1021/ac2004967 (2011).

7 Clausen, S. K., Bergqvist, M., Poulsen, L. K., Poulsen, O. M. & Nielsen, G. D. Development of sensitisation or tolerance following repeated OVA inhalation in BALB/cJ mice. Dose-dependency and modulation by the Al(OH)(3) adjuvant. *Toxicology* **184**, 51-68, doi:10.1016/s0300-483x(02)00583-8 (2003).
